# CD56 expression in breast cancer induces sensitivity to natural killer-mediated cytotoxicity by enhancing the formation of cytotoxic immunological synapse

**DOI:** 10.1038/s41598-019-45377-8

**Published:** 2019-06-19

**Authors:** Ghina Taouk, Ola Hussein, Moussa Zekak, Ali Abouelghar, Yasser Al-Sarraj, Essam M. Abdelalim, Manale Karam

**Affiliations:** 1 Cancer Research Center, Qatar Biomedical Research Institute (QBRI), Hamad Bin Khalifa University (HBKU), Qatar Foundation (QF), PO Box 34110, Doha, Qatar; 2 College of Pharmacy, Qatar University, Doha, Qatar; 3 Department of Biological Sciences, Carnegie Mellon University in Qatar, Doha, Qatar; 4 Diabetes Research Center, Qatar Biomedical Research Institute (QBRI), Hamad Bin Khalifa University (HBKU), Qatar Foundation (QF), PO Box 34110, Doha, Qatar; 5 College of Health & Life Sciences, Hamad Bin Khalifa University (HBKU), Qatar Foundation (QF), Doha, Qatar

**Keywords:** Cancer, Breast cancer

## Abstract

We examined the potential value of the natural killer (NK) cell line; NK-92, as immunotherapy tool for breast cancer (BC) treatment and searched for biomarker(s) of sensitivity to NK-92-mediated cytotoxicity. The cytotoxic activity of NK-92 cells towards one breast precancerous and nine BC cell lines was analyzed using calcein-AM and degranulation assays. The molecules associated with NK-92-responsiveness were determined by differential gene expression analysis using RNA-sequencing and validated by RT-PCR, immunostaining and flow cytometry. NK-target interactions and immunological synapse formation were assessed by fluorescence microscopy. Potential biomarker expression was determined by IHC in 99 patient-derived BC tissues and 10 normal mammary epithelial tissues. Most (8/9) BC cell lines were resistant while only one BC and the precancerous cell lines were effectively killed by NK-92 lymphocytes. NK-92-sensitive target cells specifically expressed CD56, which ectopic expression in CD56-negative BC cells induced their sensitivity to NK-92-mediated killing, suggesting that CD56 is not only a biomarker of responsiveness but actively regulates NK function. CD56 adhesion molecules which are also expressed on NK cells accumulate at the immunological synapse enhancing NK-target interactions, cytotoxic granzyme B transfer from NK-92 to CD56-expressing target cells and induction of caspase 3 activation in targets. Interestingly, CD56 expression was found to be reduced in breast tumor tissues (36%) with strong inter- and intratumoral heterogeneity in comparison to normal breast tissues (80%). CD56 is a potential predictive biomarker for BC responsiveness to NK-92-cell based immunotherapy and loss of CD56 expression might be a mechanism of escape from NK-immunity.

## Introduction

In the past decades, the advances in molecular biomarkers and the progress in treatment modalities have together contributed to improvements in breast cancer diagnosis, classification, and individualized therapy, and as a consequence in patient overall survival^1–3^. However, despite the marked decline in the breast cancer death rates over time, this disease remains the second leading cause of cancer death among women^4^. In fact, in many cases tumors do not respond to the currently available treatments or relapse after initial response^5^. Therefore, new treatment strategies are still required to eliminate these resistant tumors and improve clinical outcomes in patients. More recently, some experimental and clinical studies suggest a potential value of natural killer (NK)-cell based immunotherapy to eliminate residual breast tumor cells^3^.

NK cells are lymphocytes of the innate immune system specialized in the detection and clearance of “modified-self” cells, such as cancer and virus-infected cells, without any prior-sensitization^6^. NK cells develop mainly in the bone marrow and mature NK cells are exported to the periphery and are found in the blood, all of the lymphoid organs and some parenchymal tissues (lungs and liver)^7^. NK cells directly recognize their target through a complex array of regulatory (activating and inhibitory) receptors that monitor cell surfaces of autologous cells for an aberrant expression of self-ligands and cell stress markers, which frequently occurs in tumors^8–10^. Following NK-target recognition, NK cells adhere to their target to create an immunological synapse where the regulatory receptors spatially cluster in order to increase the efficacy of intracellular signaling^11,12^. NK activation results in the polarization of cytotoxic granules (containing cytolytic effector molecules such as granzymes and perforins) toward the immunological synapse and in their directed exocytosis. Thus, following membrane perturbation by perforin at the immune synapse, granzymes enter the target cells where they can cleave numerous substrates in the cytoplasm and nucleus leading to the induction of apoptosis^13^. Although the perforin/granzyme pathway is the main mechanism of NK-mediated cytotoxicity, NK cells can also induce apoptosis in target cells through the tumor necrosis factor (TNF) receptor pathway^14^. Furthermore, active NK cells can secrete a variety of cytokines and chemokines that might exert direct anti-tumor activity or promote innate and adaptive responses^15^.

Following the progress in understanding the biology and anti-tumoral function of NK cells, these lymphocytes have recently become a powerful cell-based cancer immunotherapy tool^16^. However, so far, the success of NK cell-based immunotherapy has largely been confined to hematological cancers^17,18^, and to a lesser extent to neuroblastoma^19^ and glioblastoma^20,21^. On the other hand, other types of cancer such as breast cancer were found to be resistant to NK cell-based therapy^3,16,22–24^.

In addition to the cancer type-dependent therapeutic potential of NK cells, other major overall challenges in NK cell-based therapy include the difficulty to obtain sufficient numbers of clinical-grade and functionally active primary NK cells for infusion in addition to the inter- and intra-donor variabilities^25,26^. Alternatively, a well-characterized cell line; NK-92, established from patient with clonal NK-cell lymphoma can be easily and reproducibly expanded from a good manufacturing practice (GMP)-compliant cryopreserved master cell bank^26–28^. NK-92 cell line is highly cytotoxic; it expresses high levels of cytolytic pathway molecules, such as perforin and granzyme B^29,30^, lacks almost all killer cell immunoglobulin-like receptors (KIRs) and expresses major activating receptors (such as NKG2D, NKp30, NKp46)^29^. NK-92 cytotoxic potency was demonstrated against a wide range of tumor types including leukemia, lymphoma, myeloma, melanoma and prostate cancer *in vitro*^30,31^. Furthermore, in large number of preclinical studies using SCID mice, infusion of the parental NK-92 cells has been shown to eliminate AML^31^, myeloma^32^ and melanoma^33^. Moreover, subsequent phase I clinical testing demonstrated safety of NK-92 infusions even at high doses and clinically significant responses were seen in a subset of patients with melanoma, lung cancer, kidney cancer and AML^26,34,35^. For these reasons, NK-92 represents an ideal tool for cancer immunotherapy; however, its cytotoxic potential towards breast cancer cells is still unknown.

The aim of this study was to analyze the cytotoxic activity of NK-92 cells towards breast cancer cells and to identify potential mechanism(s) and predictive biomarker(s) of breast cancer-sensitivity to NK-92-mediated cytotoxicity. We focused on the role of the adhesion molecule and archetypal phenotypic marker of NK cells; i.e. the cluster differentiation 56 (CD56), also known as neural cell adhesion molecule-1 (NCAM-1), which role in NK cell cytotoxic function was still unknown.

## Results

### Different susceptibilities of breast cancer cell lines and overgrowing precancerous mammary epithelial cells to NK-92-mediated cytotoxicity

**Figure 1:**
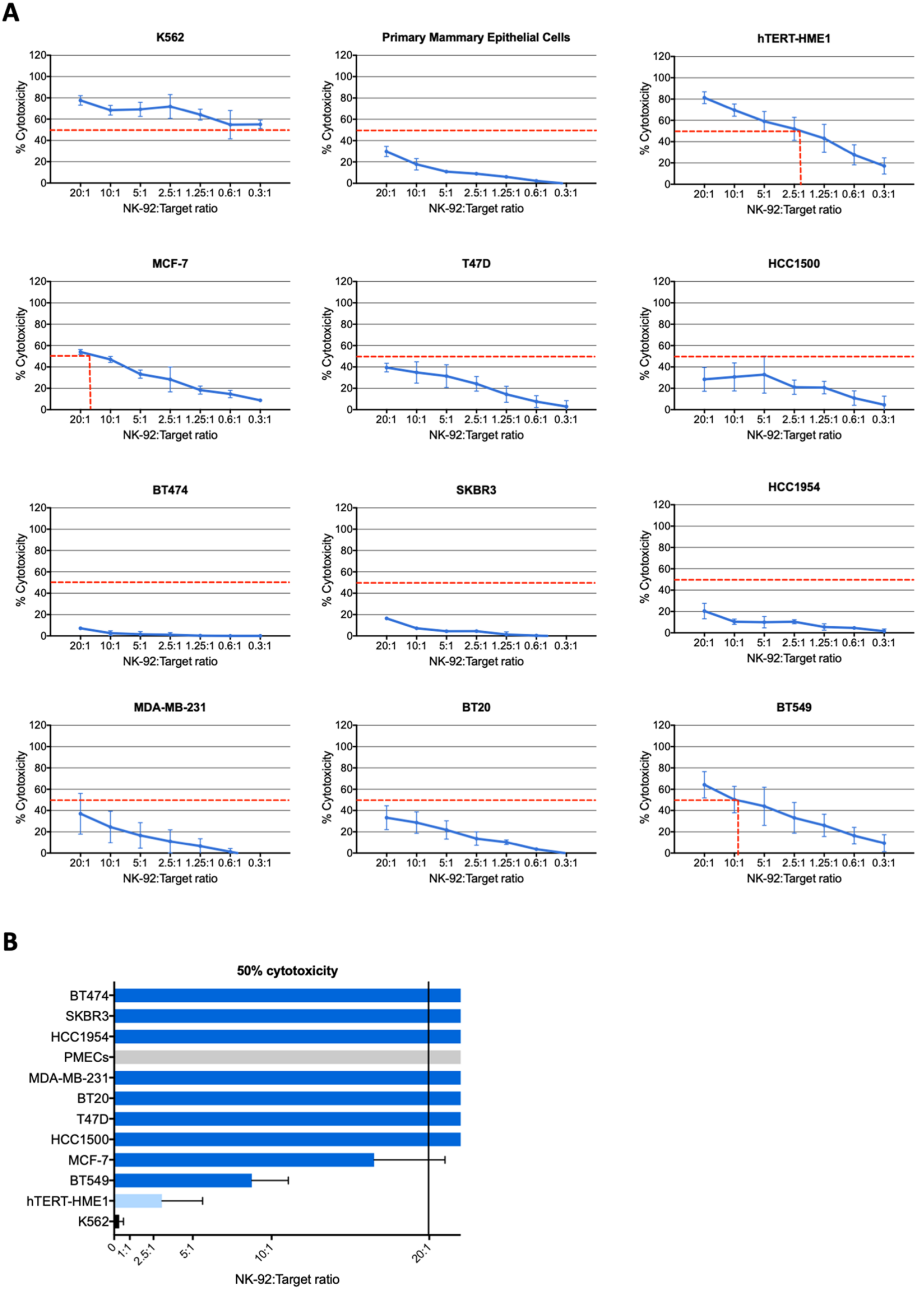
Cytotoxic activity of NK-92 cells towards breast cancer cells and mammary epithelial cells. NK-92 cells were cocultured with calcein-AM-prelabeled target cells at different NK-92:target ratios and the specific target cell lysis was determined after 4 h. (**A**) Results presented are mean percentages of cytotoxicity ± SD for three to nine independent experiments performed each in triplicates. (**B**) Bar graph showing the minimal significant NK-92:target ratios to cause lysis of 50% target cells.

To check the potential value of the NK-92 cell line as immunotherapy tool for breast cancer treatment, the cytotoxic activity of NK-92 cells was tested towards nine human breast cancer cell lines (MCF-7, T47D, HCC1500, BT474, SKBR3, HCC1954, MDA-MB-231, BT20 and BT549). A cell line of human mammary epithelial cells immortalized with the human telomerase reverse transcriptase; hTERT (hTERT-HME1) was also used as target to test the cytotoxic activity of NK-92 cells towards overgrowing but precancerous mammary epithelial cells. In fact, the acquisition of constitutive telomerase expression is a critical step during the malignant transformation of human cells. It contributes to cancer development by maintaining stable telomere length and unlimited cell proliferation but also by rendering the cells more susceptible to oncogenic transformation^36^. A normal primary human mammary epithelial cell line (PMEC) was used as negative control and the K562 leukemic cell line was used as positive control as it is known to be a highly sensitive target for NK cells^37^. As shown in Fig. 1, NK-92 cells very effectively killed K562 and hTERT-HME1 cells (50% cytotoxicity reached at very low NK-92:Target ratios ≤ 0.3:1 and 2.2:1, respectively). However, the normal mammary epithelial cells and most breast cancer cell lines (except for BT549 and MCF-7) were relatively resistant to NK-92 cytotoxicity as 50% cytotoxicity was not reached even at high NK-92:Target ratio (20:1). Although BT549 and MCF-7 breast cancer cells were responsive to NK-92 cytotoxicity, they were by far less sensitive than the hTERT-immortalized mammary epithelial cells (50% cytotoxicity reached at NK-92:Target ratios of ~8:1 and 17:1, respectively vs 2.2:1).

### The high sensitivity of hTERT-immortalized mammary epithelial cells and BT549 breast cancer cells to NK-92-mediated cytotoxicity is associated with enhanced NK-92 degranulation

**Figure 2:**
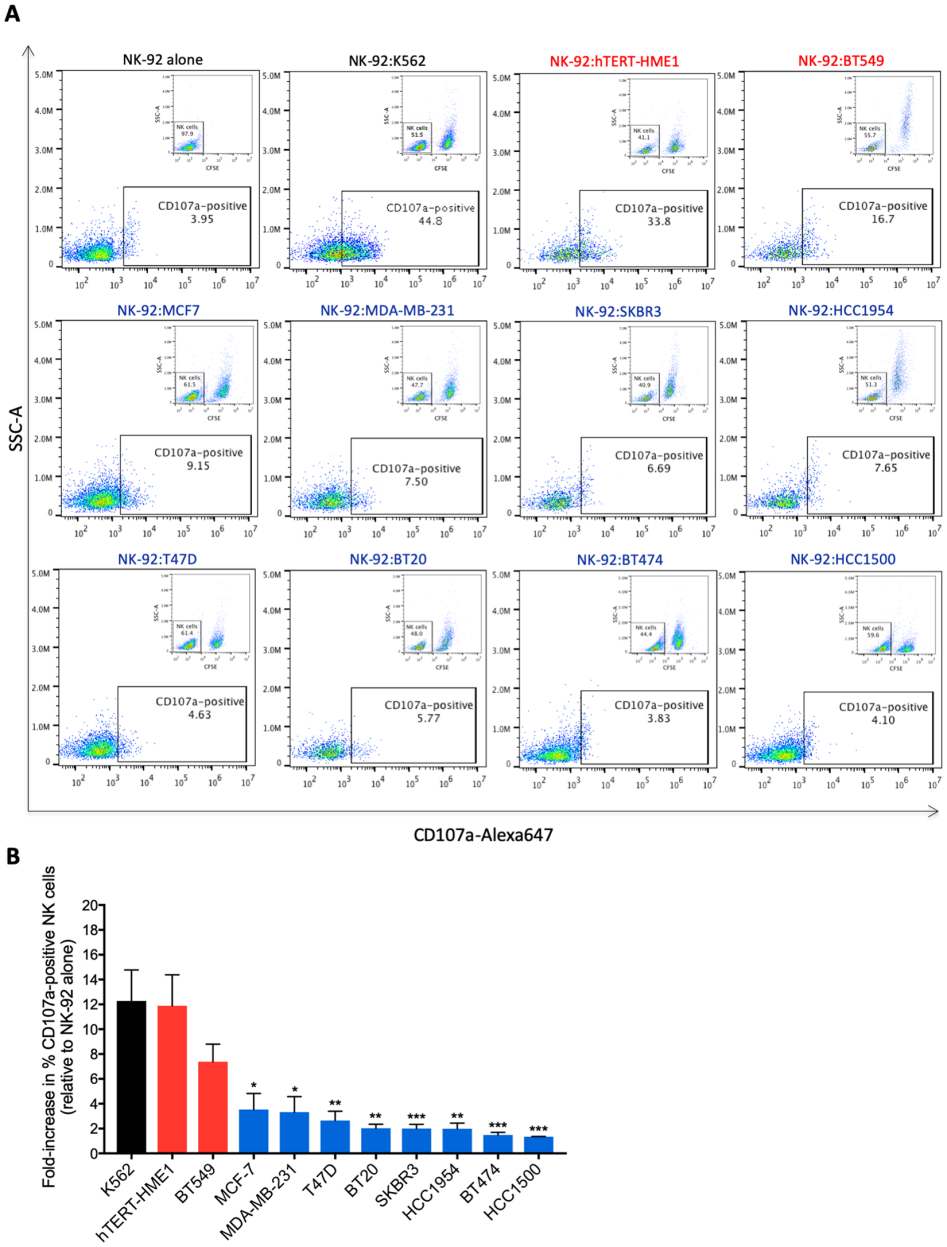
Degranulation state of NK-92 cells after coculture with breast cancerous and precancerous cells. NK-92 cells were cocultured with CFSE-labeled NK-92-sensitive (red) and NK-92-resistant (blue) target cells at 1:1 ratio for 2 h. Next, CD107a expression on the surface of NK-92 cells was assessed by flow cytometry. (**A**) Representative experiments of at least three independent experiments showing the percentage of CD107a-positive NK-92 cells. (**B**) Graph presenting the fold-increase in target-induced CD107a-positive NK-92 cells (relative to spontaneous CD107a expression) ± SD for at least three independent experiments. *p < 0.05; **p < 0.01; ***p < 0.001 versus BT549 cells.

To gain insight into the mechanism of increased sensitivity of hTERT-HME1 and BT549 cells to NK-92-mediated lysis, we first checked whether these target cells could better induce NK activation in comparison to the NK-92-resistant breast cancer cells, rather than being only more sensitive to apoptotic death. Therefore, following coculture with the different target cells, CD107a expression on the surface of NK-92 cells (a hallmark of NK activation and degranulation)^38^ was assessed by flow cytometry (Fig. 2). Results showed approximately 12-, 11- and 7-fold increase in CD107a expression on the surface of NK-92 cells after 2 h coculture with K562, hTERT-HME1 and BT549 target cells, respectively in comparison to NK-92 cultured without target cells (Fig. 2A,B). When cocultured with the NK-92-resistant (MDA-MB-231, SKBR3, HCC1954, T47D, BT20, BT474 and HCC1500) or the less sensitive (MCF-7) breast cancer cells, target-induced NK-92 degranulation was very low (≤3.5-fold increase in CD107a surface expression) (Fig. 2A,B). These findings suggest that the NK-92-sensitive hTERT-immortalized epithelial cells (hTERT-HME1) and cancer cells (BT549) of mammary origin as for the K562 leukemic cells are more potent in inducing NK-92 cell degranulation compared to the relatively more resistant breast cancer cells, which can be the consequence of an increased NK recognition and activation by these target cells.

### Specific association of CD56 expression in breast precancerous and cancerous cells with high sensitivity to NK-92-mediated killing

Target cells are recognized by NK cells and can induce the activity of these effectors through regulatory receptors/ligands and adhesion molecules expressed on their surface^8,39^. Thus, such molecules might be involved in conferring the high sensitivity of hTERT-HME1 and BT549 to NK-92-mediated cytotoxicity.

**Figure 3:**
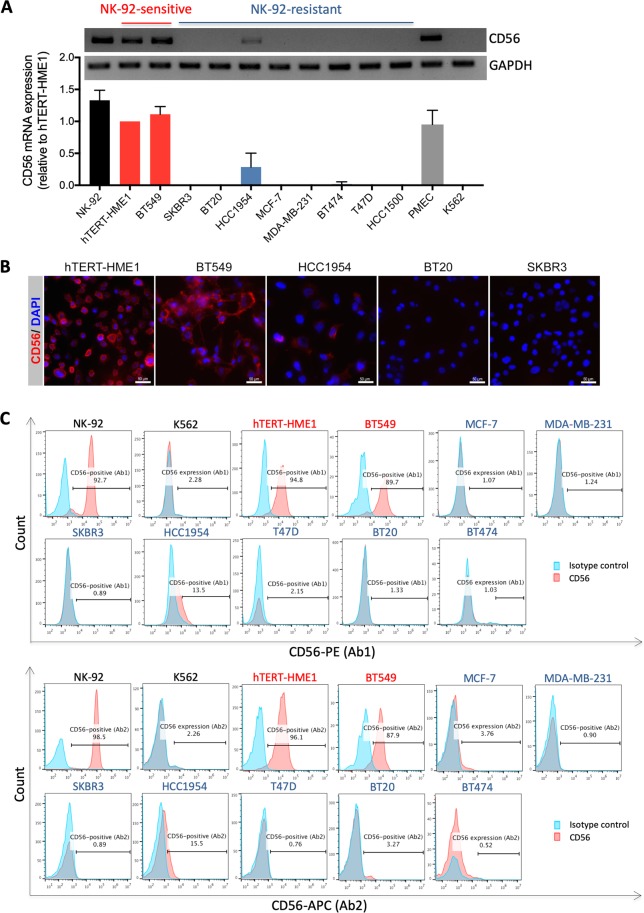
Specific expression of CD56 adhesion molecule on breast precancerous and cancerous cells exhibiting high sensitivity to NK-92-mediated killing. (**A**) Analysis of CD56 mRNA expression in NK-92, hTERT-immortalized epithelial mammary cells (hTERT-HME1) and breast cancer cells (BT549, SKBR3, BT20, HCC1954, MCF-7, MDA-MB-231, BT474, T47D and HCC1500) by RT-PCR. The expression of GAPDH gene was used as housekeeping control. The RT-PCR agarose gel electrophoresis images are those of a typical experiment and the results presented in the corresponding densitometry bar graph are the means ± SD for three independent experiments. (**B**) Representative immunofluorescence images of NK-sensitive (hTERT-HME1 and BT549) and NK-resistant (HCC1954, BT20 and SKBR3) target cells that were fixed, permeabilized and stained for CD56 and nuclei (DAPI). (**C**) Analysis of CD56 protein expression on the surface of viable NK-92 cells and the indicated target cells by flow cytometry using two different antibodies (Ab1 and Ab2) that recognize different epitopes on the extracellular portion of this protein. The histograms presented indicate the percentage of CD56-positive cells and are representative results from two independent experiments.

In a first attempt to analyze NK-92 degranulation (CD107a surface expression) following coculture with breast cancer target cells, we used, in association with CD107a staining, CD56 (the archetypal phenotypic marker of NK cells) staining to discriminate between NK-92 (CD56-positive) and breast cancer cells (expected to be CD56-negative) (Supplementary Fig. 1A). The results showed that when NK-92 cells were cultured without target cells, all the cells (>99.8%) were CD56-positive, as expected. When NK-92 cells were cocultured with NK-92-resistant cells at NK-92:target ratio of 1:1, about half of the population was CD56-positive (corresponding to NK-92 cells) and the other half was CD56-negative (corresponding to breast cancer cells). However, unexpectedly, when NK-92 cells were cocultured with NK-92-sensitive cells, almost all the cells (>95%) were CD56-positive, suggesting that the NK-92-sensitive breast cancer cells would express the adhesion molecule CD56. Consistently, differential gene expression analysis by RNA-sequencing revealed that CD56 mRNA is indeed highly and specifically expressed in NK-92-sensitive cells (hTERT-HME1 and BT549) but absent (SKBR3, BT20, MCF-7, MDA-MB-231, BT474, T47D and HCC1500) or very faintly expressed (HCC1954) in the NK-92-resistant breast cancer cells (Supplementary Fig. 1B). The specific expression of CD56 mRNA in these NK-92-sensitive cells was validated by RT-PCR (Fig. 3A) and confirmed at the protein level by immunofluorescence (Fig. 3B).

Next, as the expression of CD56 at the surface of target cells is critical for its role in cell adhesion, we checked its presence at the surface of NK-92-sensitive target cells by flow cytometry which was performed on viable, non-permeabilized cells and by using two different antibodies that bind to different epitopes in the extracellular domain of CD56 (Fig. 3C). Results showed that CD56 is expressed at the surface of approximately 95.45% and 88.8% of the NK-92-sensitive hTERT-HME1 and BT549 cells, respectively. In contrast, among the NK-92-resistant breast cancer cells, only a small portion of the HCC1954 cell line (approximately 14.5%) express CD56 on their surface while all other resistant cells (MCF-7, MDA-MB-231, SKBR3, T47D, BT20 and BT474) are negative for CD56 protein expression. Furthermore, as expected most NK-92 cells (approximately 95.6%) expressed CD56 on their surface. Interestingly, in contrast to the NK-92-sensitive hTERT-immortalized epithelial and cancer cells of mammary origin, the leukemia cell line K562 didn’t express CD56 (Supplementary Fig. 1B, Fig. 3A,C). Furthermore, the human primary mammary epithelial cells (PMECs) also expressed CD56 (Supplementary Fig. 1B and Fig. 3A). Taken together, these results show for the first time that CD56 adhesion molecule can be expressed in a subset of normal, precancerous and cancerous cells of mammary origin and suggest its strong association with sensitivity to NK-92-mediated killing, which seems to be tissue type-specific.

### Increased expression of CD56 at the immunological synapse and association with cytotoxic granzyme B transfer from NK-92 into target cell and caspase 3 activation

**Figure 4:**
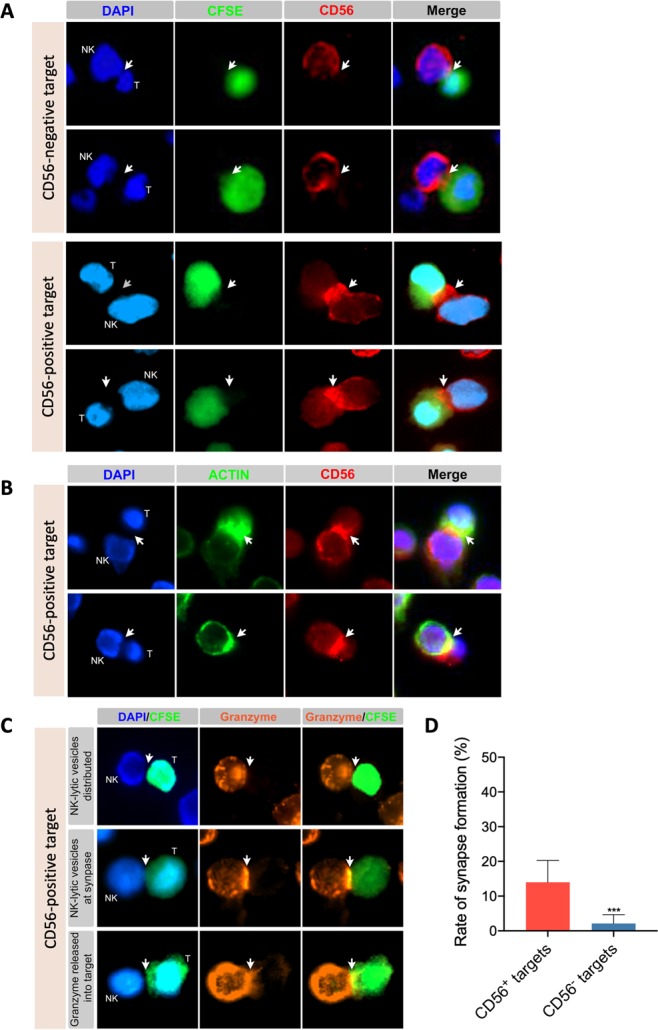
CD56 expression at immunological synapse and association with granzyme B transfer from NK-92 into CD56-positive target cells. CD56-expressing (hTERT-HME1) and/or CD56-negative (MCF-7, MDA-MB-231 and/or SKBR3) target cells were labeled (panels A and C) or not (panel B) with CFSE and cocultured with NK-92 cells at 1:1 effector-to-target ratio for 2 hours. Cells were then transferred to poly-L-lysin-coated coverslips, fixed, permeabilized and stained as indicated for CD56 (panels A and B), actin (panel B), granzyme b (panel C) and nuclei (DAPI) (panels A, B and C) before analysis by fluorescent microscopy. Images is panel A are for MCF-7 and MDA-MB-231 (CD56-negative targets) and hTERT-HME1 (CD56-positive target) and correspond to representative fields from two or three independent experiments. Images in panel C are for three different synapses observed after 2 hours in the same coculture of NK-92 and hTERT-HME1. (**D**) Graph presenting the mean rates of synapse formation ± SD obtained from six representative fields for CD56-positive target (hTERT-HME1) and nine representative fields for CD56-negative targets (three fields for each cell line MCF-7, MDA-MB-231 and SKBR3). ***p < 0.001.

Next, we checked whether CD56 expression in target cells is merely a marker of sensitivity to NK-92-mediated cytotoxicity or whether it is actively involved in the immune effector function. As CD56 is an adhesion molecule that can regulate cellular function through homophilic interactions^40^, we hypothesized that CD56 molecules on NK-92 cells and those on target cells would interact together to enhance the formation and stabilization of the immune synapse between these effector and target cells. To test this hypothesis, CD56-negative (MCF-7, MDA-MB-231 and SKBR3) and CD56-expressing (hTERT-HME1) target cells were labeled with CFSE and cocultured with NK-92 cells at 1:1 effector-to-target ratio for 2 hours before immunostaining for CD56 and fluorescent microscopy analysis. Results showed that the rate of immune synapse formation is significantly higher when NK cells are co-cultured with CD56-positive target cells (13.99%) than CD56-negative target cells (2.12%) (Fig. 4D). Thus, CD56 expression in target cells associates with enhanced immune synapse formation and/or stabilization.

Analysis of the localization of CD56 within the NK-target conjugates showed that this adhesion molecule strongly accumulates at the effector-target intercellular contacts specifically when target cells are CD56-positive but not when they are CD56-negative (Fig. 4A). Next, to further confirm that these interactions correspond to the immunological synapse the cocultures of NK-92 and target cells were co-stained for actin (to visualize actin cytoskeleton polymerization at immune synapse^41^; and CD56 then analyzed by fluorescent microscopy (Fig. 4B). Results showed that indeed CD56 molecules strongly accumulate at the immune synapse, which was accompanied by granzyme B cytotoxic molecule transfer from NK-92 to the CD56-expressing target cells (Fig. 4C).

**Figure 5:**
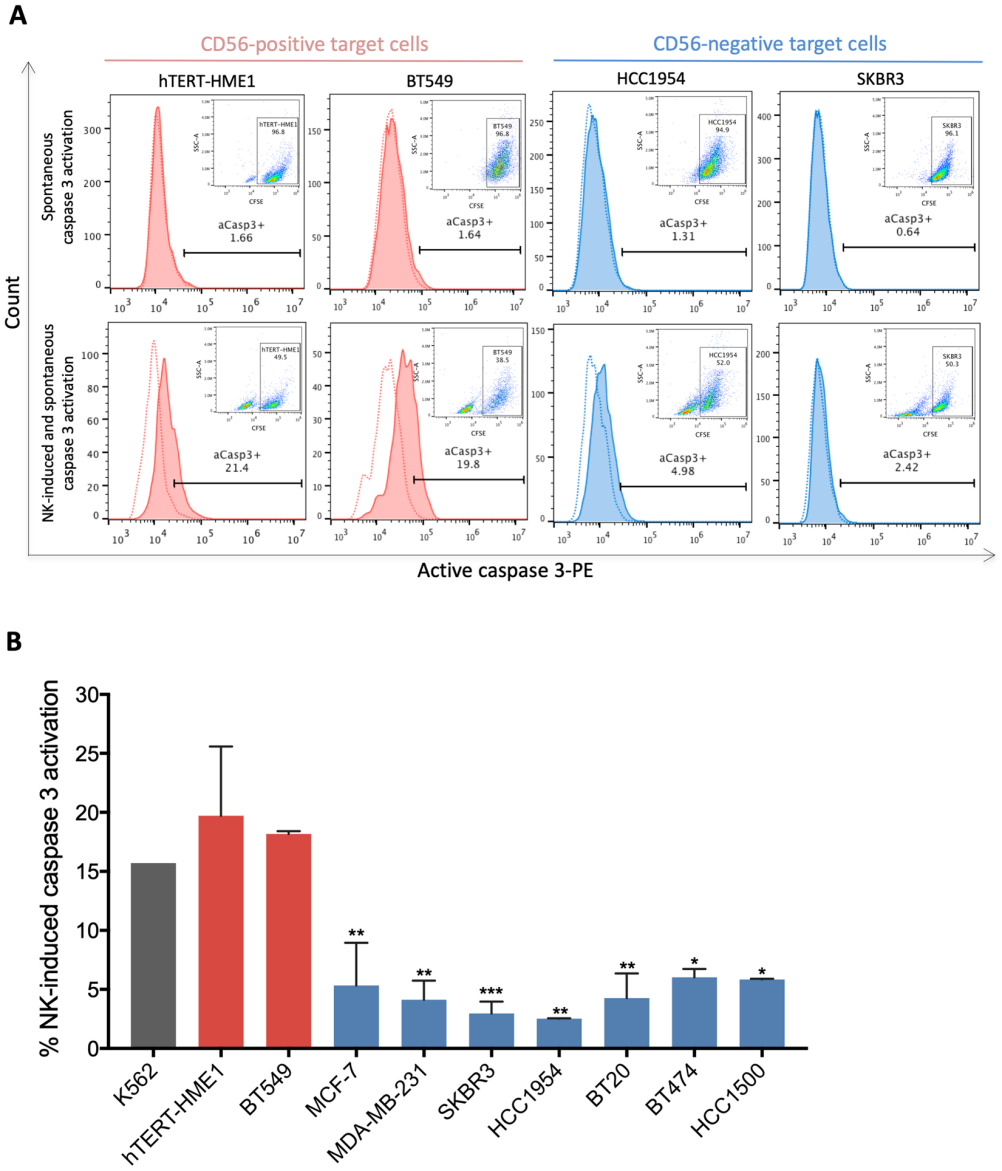
Association of CD56 expression in target cells with increased NK-92-induced caspase 3 activation. NK-92 cells were coculture or not with CFSE-labeled target cells for 2 hours at 1:1 effector-to-target ratio then NK-induced and spontaneous active caspase 3 levels in target cells were measured by flow cytometry. (**A**) Representative histograms showing the percentage of active caspase 3-positive (aCasp3+) target cells. (**B**) Graph presenting the mean percentages ± SD of NK-induced caspase 3 activation (corrected for spontaneous caspase 3 activation) in target for at least three independent experiments. *p < 0.05; **p < 0.01; ***p < 0.001 versus hTERT-HME1 target cells.

Following their delivery into the target cells at immune synapse, granzymes can induce apoptosis by different pathways^13^. A common factor between these granzyme-induced apoptosis pathways, pro-caspase 3 is the main caspase that will be cleaved and activated downstream of granzyme B, which is thought to be a critical first step in cytotoxic lymphocyte-induced apoptosis^13^. Therefore, to further confirm the association of CD56 expression in target cells with enhanced activation and cytotoxic function of NK-92 cells, active caspase 3 levels in target cells cocultured or not with NK-92 cells were measured by flow cytometry (Fig. 5). Our results showed that while active caspase 3 was absent in almost all of the target cells (98.34–99.36%) (Fig. 5A, upper plots), following coculture with NK-92, it was strongly increased in CD56-positive target cells (19.8–21.4% active caspase 3-positive cells) but not in CD56-negative target cells (2.42–4.98% active caspase 3-positive cells) (Fig. 5A). Overall, NK-92-induced caspase 3 activation was 3.13 to 7.5-fold higher in CD56-positive target cells in comparison to CD56-negative target cells (Fig. 5B). Taken together, these results indicate that CD56 strongly accumulates at the immunological synapse and associates with cytotoxic granzyme B transfer from NK-92 into CD56-positive target and with a higher rate of NK-92-induced apoptosis. Thus, target-expressed CD56 might be involved in the formation and stabilizing of the immunological synapse allowing enhanced NK activation and stimulation of target cell death.

### Induction of CD56 expression in breast cancer cells enhances their sensitivity to NK-92-mediated cytotoxicity

**Figure 6:**
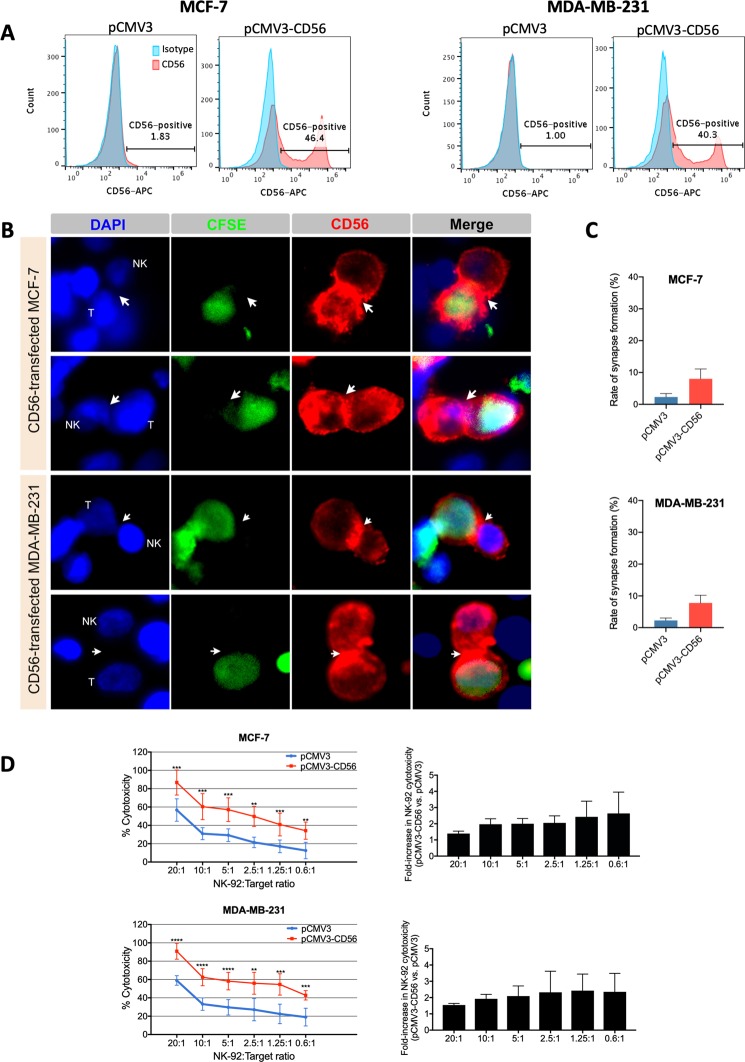
Induction of CD56 expression in breast cancer cells enhances their sensitivity to NK-92-mediated cytotoxicity. CD56-negative MCF-7 and MDA-MB-231 parental cells were transiently transfected with either pCMV3-CD56 plasmid or pCMV3 empty plasmid. After 48 hours, the transfected cells were assessed for CD56 expression by flow cytometry (panel A), for the rate of immune synapse formation and CD56 localization by CFSE-labeling, coculture with NK-92 cells at 1:1 effector-to-target ratio for 2 h and immunofluorescence microscopy (panel B and C) and for their responsiveness to NK-92-mediated cytotoxicity by calcein-AM cytotoxicity assay (panel D). (**A**) The histograms presented indicate the percentage of CD56-positive cells and are representative results from at least five independent experiments. (**B**) Fluorescence images are representative fields from two independent experiments. (**C**) Graphs presenting the mean rates of synapse formation ± SD obtained from two full slides for each condition. (**D**) Results presented are means ± SD for three independent experiments performed each in triplicates. **p < 0.01; ***p < 0.001; ****p < 0.0001.

In order to check the potential direct NK-sensitizing role of CD56 in breast cancer cells, this adhesion molecule was transiently overexpressed in CD56-negative MCF-7 and MDA-MB-231 cells. Then, the potential localization of CD56 at the immune synapse and its role in target responsiveness to NK-92-mediated cytotoxicity were analyzed by immunofluorescence microscopy and calcein-AM cytotoxicity assay, respectively (Fig. 6). We found that after two days of transfection with CD56 containing plasmid (pCMV3-CD56), around 40.3%-46.4% of breast cancer cells expressed CD56 (Fig. 6A) that was strongly concentrated at the immune synapse between NK-92 and target cells (Fig. 6B). Furthermore, the rate of synapse formation (Fig. 6C) and the percentage of NK-92-mediated cytotoxicity (Fig. 6D) were strongly increased (by approximately 3.4-fold and 2.11-fold, respectively) for both CD56-transfected MCF-7 and MDA-MB231 cells in comparison to empty vector-transfected control cells. Thus, these results validate the role of CD56 in immune synapse formation and/or stabilization and in increasing the sensitivity of breast cancer cells to NK-92-mediated cytotoxicity.

### Analysis of CD56 expression in patient-derived breast cancer tissues and association with clinicopathological features and patient survival

The data presented above suggests that breast tumors expressing CD56 might be more sensitive to NK-mediated cytotoxicity and thereby these patients might be potential candidates for NK-92 cell-based immunotherapy. However, to our knowledge, CD56 expression in breast tissues was not previously reported. Therefore, to get some insight into the potential relevance of CD56 as predictive biomarker for NK-sensitivity in breast cancer, we analyzed its expression status in breast cancer tissues using breast carcinoma microarray, containing 100 cases of invasive ductal carcinoma and 10 adjacent normal breast tissues (Supplementary Fig. 2 and Table 1). The immunohistochemical analysis showed that 24.2% of tumor tissues expressed high levels of CD56, 12.1% expressed moderate levels of CD56, and 63.7% expressed very low levels or were completely negative for CD56 (Table 1). Noteworthy, 80% (8 out of 10) of the normal breast tissues expressed CD56 (Supplementary Fig. 2), consistently with the primary mammary epithelial cell line (PMEC) (Fig. 3A and Supplementary Fig. 1B). However, in contrast to breast tumor tissues that mostly expressed low levels of CD56, 60% of breast normal tissues were found to express high levels of CD56, 10% expressed moderate levels of CD56 and 30% expressed very low levels of CD56 (Supplementary Fig. 2).

**Table 1:** Immunohistochemical analysis of CD56 expression in breast cancer tissues and relation to standard clinicopathological factors.

	Total number of tumors (%)	Number of tumors (%)		*P* ^b^
		High to moderate CD56 expression^a^ (30–100%)	Low and absent CD56 expression^a^ (0–29%)	
*Total*	99 (100%)	36 (36.4%)	63 (63.7%)	
*Age*				0.52 (NS)
<60	87 (87.9%)	31 (86.1%)	56 (88.9%)	
≥60	12 (12.1%)	5 (13.9%)	7 (11.1%)	
Tumor size				0.68 (NS)
≤2 cm	10 (10.1%)	3 (8.3%)	7 (11.1%)	
2–5 cm	61 (61.6%)	22 (61.1%)	39 (61.9%)	
>5 cm	28 (28.3%)	11 (30.6%)	17 (27%)	
*Lymph node status*				
Negative	58 (58.6%)	24 (66.7%)	34 (54%)	0.06 (NS)
Positive	41 (41.4%)	12 (33.3%)	29 (46%)	
*Tumor grade* ^c^				
I	0 (0%)	0 (0%)	0 (0%)	0.44 (NS)
II	54 (56.8%)	18 (51.4%)	36 (60%)	
III	41 (43.2%)	17 (48.6%)	24 (40%)	
*ER status* ^d^				
Negative	42 (42.9%)	15 (41.7%)	26 (42.6%)	0.89 (NS)
Positive	56 (57.1%)	21 (58.3%)	35 (57.4%)	
*PR status* ^e^				
Negative	62 (63.9%)	23 (63.9%)	39 (63.9%)	>0.99 (NS)
Positive	35 (36.1%)	13 (36.1%)	22 (36.1%)	

Breast cancer tissue microarray used: BC081120c (US Biomax).

^a^High expression (70–100% of CD56-positive cells per tissue), moderate expression (30–69% of CD56-positive cells per tissue) and low expression (0–29% of CD56-positive cells per tissue); ^b^χ^2^ Test; ^c^Information available for 95 patients; ^d^Information available for 98 patients; ^e^Information available for 97 patients.

Next, the possible relationship between CD56 expression and clinicopathological variables that were available for the microarray was determined (Table 1). However, no relationship was found between the expression pattern of CD56 and all available classical pathological and clinical parameters (i.e. patient age, tumor size, lymph node status, tumor grade, ER status and PR status).

Finally, to check whether CD56 mRNA expression might influence prognosis in breast cancer patients, we took advantage of the KM plotter platform that uses gene expression data and patient survival information downloaded from GEO, EGA and TCGA. The Kaplan-Meier survival plots showed that high mRNA expression of CD56 significantly associates with better relapse-free survival (RFS) but not overall survival (OS) (Supplementary Fig. 3A). However, as this is data from bulk mRNA, it cannot be distinguished whether the higher CD56 expression is actually due to higher expression on tumor cells or whether it is due to greater infiltration of NK cells. Therefore, to control for the presence of NK cells within the tumors, the prognostic value of another NK marker; NKp46, was also checked and found to be significantly associated with better RFS and OS (Supplementary Fig. 3B). In contrast to CD56, NKp46 was not found to be expressed in breast tumor tissues (Supplementary Fig. 4) or cell lines (Supplementary Fig. 3C). Thus, together, this data suggests that the good prognostic value of NKp46 would reflect the prognostic value of NK cell infiltration in breast tumors and therefore, the good prognostic value of CD56 mRNA expression in breast tumor tissues might be at least in part due to the enhanced infiltration by NK cells and not necessarily related to the expression of this adhesion molecule in tumor cells themselves.

## Discussion

In the present study, we checked the potential value of the NK-92 cell line as immunotherapy tool for breast cancer treatment and searched for biomarkers and mechanisms of sensitivity to NK-mediated cytotoxicity. To our knowledge, the cytotoxic potency of NK-92 cells had never been determined against breast cancer cell lines; nonetheless, it was widely analyzed against leukemia cell lines, which were found to be all highly sensitive (more than 50% cytotoxicity at very low effector:target ratios; less than 1:1 for most cell lines and less than 9:1 for the least sensitive ones)^31^. Consistently, our results showed that K562 leukemia cells, used as positive control, are highly sensitive to NK-92-mediated lysis (50% cytotoxicity at effector:target ratio less than 1:1). On the other hand, almost all breast cancer cell lines (except for 1 out of 9) were found to be resistant to NK-92 cells (50% cytotoxicity was not reach at effector:target ratio of 9:1). This *in vitro* data reflects the differences in NK cell-based immunotherapy clinical outcomes, which were successful in hematological cancers^17,18^, but not in breast cancer^3,16^. In addition to the breast cancer cells, we determined the cytotoxic activity of NK-92 lymphocytes against normal mammary epithelial cells and hTERT-immortalized mammary epithelial cells. Whereas normal mammary epithelial cells were resistant to NK-92-mediated cytotoxicity, their hTERT-immortalized counterparts were highly sensitive. The increased sensitivity of hTERT-immortalized mammary epithelial cells to NK-92-mediated lysis could be the consequence of the possible expression of classical ligands for NK-activating receptors that might probably be induced by the cellular stress caused by telomerase constitutive expression (data not shown; manuscript in preparation). For example, the differential expression analysis of NK regulating genes between the hTERT-immortalized mammary epithelial cells (hTERT-HME1) and the normal primary mammary epithelial cells (PMEC) showed that the NK-activating ligand; CD86, could be a candidate gene for such hypothesis (Supplementary Fig. 5A). In fact, CD86 seems to be expressed in hTERT-HME1 but not in PMEC. However, the expression of this stress ligand doesn’t seem to be sufficient for the induction of the sensitivity of breast precancerous/cancerous cells to NK-92-mediated cytotoxicity as it was also found to be expressed in the NK-92-resistant breast cancer cell line HCC1954 (Supplementary Fig. 5A). This hypothesis still needs to be investigated. Thus, another factor would be responsible for the difference in the responsiveness of breast cancer cells to NK-92-mediated cytotoxicity. Independently of the nature of this factor, these observations further support, in breast cancer, the previously described concept that NK cells eliminate abnormal (highly proliferative and stressed) cells to prevent cancer development while saving normal tissues and that the acquisition, by cancer cells, of mechanisms of escape from immune surveillance notably by NK cells allows cancer progression^42–44^.

The *in vitro* experimental model used in the present study, which consists of direct NK-92 and target cell co-culture, considers the tumor cell-intrinsic mechanism(s) involved in the resistance of breast cancer to NK-mediated cytotoxicity, but doesn’t take into account the regulatory effect of the tumor microenvironment^45^. Few studies have examined the tumor cell-intrinsic mechanisms of NK-escape in breast cancer. These mechanisms include: (1) the modulation of the expression of molecules involved in NK recognition and activation (i.e. increased expression of ligands for NK inhibitory receptors and/or decreased expression of ligands for NK activating receptors on target cells)^46–48^, (2) the secretion of soluble inhibitory factors that alter the function of NK cells^22,24^ and/or (3) the development of resistance to apoptosis^23^. In our experiments, the target cell supernatants, which might contain any potential NK-inhibitory soluble factors, were replaced by fresh media before coculture with NK cells; therefore, the secretion of NK-inhibitory factors by NK-92-resistant breast cancer cells might not be responsible for the observed resistance. Moreover, since our results showed an association of the decreased responsiveness to NK-mediated cytotoxicity with decreased NK degranulation (i.e. activation), our study favors the first above-mentioned mechanism of breast cancer escape from NK cells over the third one (i.e. resistance of target cells to NK-induced apoptosis). Thus, taken together, these observations suggested that molecules responsible for NK recognition and/or activation are deregulated in the two NK-92-sensitive cell lines (hTERT-HME1 and BT549) in comparison to the eight NK-92-resistant breast cancer cell lines, which we next tried to uncover.

Comparative gene expression analysis showed a specific expression of CD56 mRNA and protein only in the NK-92-sensitive (hTERT-HME1 and BT549), but not in the NK-92-resistant breast cancer cell lines (BT474, SKBR3, HCC1954, MDA-MB-231, BT20, T47D, HCC1954 and MCF-7). This observation implies that CD56 is a potential predictive biomarker of breast cancer sensitivity to NK-92-mediated cytotoxicity, which seems to be tissue specific as the highly sensitive K562 leukemia cells didn’t express this marker.

The value of CD56 as predictive biomarker for NK-92-based immunotherapy in breast cancer depends on the actual expression of this protein in patient-derived breast tissues, which had never been previously described. Physiologically, CD56 (alias neural cell adhesion molecule) is known to be abundantly expressed in the developing as well as in the adult human nervous system and plays a pivotal role in neurogenesis, neuronal migration, and neurite outgrowth^49–51^. Furthermore, CD56 is considered the archetypal phenotypic marker of NK cells^52,53^, but has also been detected on other lymphoid cells, including a subset of T cells and dendritic cells^54–56^. Therefore, to gain insight into the status of CD56 in breast cancer tissues, we analyzed the expression of this protein by IHC in 99 patient-derived breast cancer tissues and revealed high inter- and intratumoral heterogeneity regarding CD56 expression. Thus, based on our hypothesis that CD56 is a predictive biomarker for responsiveness to NK-92-mediated cytotoxicity, if adoptive NK-92 transfer is to be used in breast cancer patients, we would expect, unresponsiveness in more than two-thirds of the cases (no or very low % of CD56 expressing cells), and at best, very low responsiveness with rapid relapse in 12.1% of the cases (high intratumoral heterogeneity regarding CD56 expression; 30–69% CD56-positive cells) and better responsiveness with longer RFS in 24.2% of the cases (70–100% of CD56-positive cells per tissue). However, these expectations are very optimistic as they don’t take into consideration the accessibility of NK-92 cells to the tumor cells (even if administered after surgical removal of the breast tumor) neither the regulatory effect of the tumor microenvironment which might lower the immunotherapeutic potential of these lymphocytes^45^. Therefore, preclinical studies using patient-derived breast cancer xenograft-mouse models would allow the verification of whether the high cytotoxic activity of NK-92 cells against CD56-positive breast cancer cells is maintained *in vivo* and the identification of potential combinatory treatment in addition to NK-92 adoptive transfer to counteract any possible inhibitory effect of the tumor microenvironment.

Noteworthy, we also found that 80% (8 out of 10) of the normal breast tissues express CD56, consistently with the primary mammary epithelial cell line (PMEC). Thus, this study describes for the first time CD56 expression in tissues of mammary origin and suggests that the loss of CD56 expression might be a mechanism of breast cancer escape from NK-immunity. Interestingly, high mRNA expression of CD56 significantly associated with better prognosis.

However, the good prognostic value of CD56 mRNA expression in breast tumor tissues might be at least in part due to the enhanced infiltration by NK cells and not necessarily related to the expression of this adhesion molecule in tumor cells themselves.

The role of CD56 as predictive biomarker for NK-92-based immunotherapy could also be valid for other types of cancer in addition to breast cancer. In fact, among the solid tumor cancers, the success of NK cell-based immunotherapy has been limited to neuroblastoma and glioblastoma^16,57,58^. As these tumors are of neurectodermal origin and thereby strongly express CD56 molecules^59,60^, we can hypothesize that CD56 expression might account for the responsiveness of these tumors to NK-based immunotherapy, which needs to be experimentally tested.

In addition, to its role as predictive biomarker of responsiveness to NK-92-mediated cytotoxicity, we found that CD56 plays an active role in enhancing the sensitivity of breast cancer cells to these lymphocytes. In fact, the ectopic expression of this adhesion molecule in CD56-negative breast cancer cells enhanced their sensitivity to NK-92-mediated killing. Consistently with our finding that CD56 molecules expressed on target cells are functionally involved in inducing NK cell-mediated cytotoxicity; on the other hand, CD56 molecules on NK cells were found to play a similar role. In fact, a study by Nitta *et al*. showed that neutralization of CD56 molecules expressed on NK cells inhibits the cytotoxicity of these lymphocytes only towards target cells that express CD56 (i.e. tumor cell lines of neuroectodermal origin) but not towards CD56-negative leukemic targets^61^. Together, this finding and ours suggest that both CD56 molecules expressed on NK cells and on target cells are involved in enhancing NK function through homotypic interactions, consistently with the homotypic binding of this adhesion molecule demonstrated in neural and muscle tissues^40^. In agreement with this hypothesis, we found that CD56 molecules on NK-92 cells and those on CD56-positive target cells interact together to enhance the formation and stabilization of the immunological synapse. Furthermore, the accumulation of CD56 molecules at the immunological synapse was accompanied by cytotoxic granzyme B transfer from NK-92 to the CD56-expressing target cells and by induced caspase 3 activation in target cells. However, this resulting polarization of cytotoxic granules, transfer of granzymes into target cells and induction of apoptosis can be the consequence of either the spatial clustering of the NK regulatory receptors at the CD56-mediated interaction between NK-92 and target cells thereby increasing the efficacy of intracellular signaling^11,12^ or the direct CD56-mediated regulation of signaling pathways that control lytic granule polarization at the immune synapse. Although the role of CD56 in the latter process was not previously described, CD56 is known to regulate molecules such as fyn that are essential for promoting lytic granule polarization to the lytic synapse^40,62^. Furthermore, comparison of the differential gene expression of all known-to-date ligands for NK regulatory receptors^63^ between the different NK-92-sensitive and resistant breast precancerous and cancerous cell lines showed that three ligands CD70, CD72 and COL3A1 have an expression profile comparable to that of CD56 (Supplementary Fig. 5A). Among these, only CD72 could be involved in the increased sensitivity of these target cells to NK-mediated cytotoxicity. In fact, on the one hand Col3A1 is a NK-inhibitory molecule and on the other hand the receptor for CD70 (i.e. CD27) is not expressed in NK-92 cells (Supplementary Fig. 5B). However, even if the NK-activating ligand, CD72, whose corresponding receptor (SEMA4D) is expressed by NK-92 cells (Supplementary Fig. 5B) might play a role in inducing breast cancer sensitivity to NK-mediated cytotoxicity (a hypothesis that needs to be tested), the role of CD56 in this same mentioned process is independent of the expression CD72. In fact, as shown in the present study, the induction of CD56 expression in MCF-7 and MDA-MB-231 which are CD72-negative strongly enhanced their sensitivity to NK-mediated cytotoxicity.

Whatever the exact mechanism involved, our observations strongly suggest that CD56 expression on breast cancer cells enhances their susceptibility to NK-92 mediated cytotoxicity and therefore induction of its expression in CD56-negative cells might become a novel attractive approach for sensitizing initially resistant breast tumors.

Although in this present study we focused on the NK-92 cell line, which presents several advantages over the other sources of NK cells^26^, our conclusions might also be applicable on other types of NK cells (such as peripheral blood NK cells or stem cell-derived NK cells) since they also express CD56. However, in contrast to NK-92 cells, normal NK cells express the NK inhibitory receptors; KIRs^64^. Therefore, further experiments using other sources of NK cells could be performed to address whether the NK-activating role of CD56 molecules on target cells could counteract the KIR-mediated inhibitory signals, which is not concerning in the case of NK-92 cells that lack most KIRs^64^.

In conclusion, this study identified CD56 as predictive biomarker for breast cancer sensitivity to NK-92-mediated cytotoxicity and as new regulator of the NK-immune function by enhancing the interaction between NK and breast cancer cells and thereby inducing immune synapse formation and stabilization. Thus, NK-92 cell line is a potential effective immunotherapy tool for CD56-positive breast cancer treatment and induction of CD56 expression combined with NK-92-adoptive transfer might become a novel attractive approach for sensitizing initially resistant CD56-negative breast tumors.

## Methods

### Cell culture

All human breast tissue derived cell lines (hTERT-HME1, PMEC, BT20, BT474, BT549, HCC1500, HCC1954, MCF-7, MDA-MB-231, SKBR3 and T47D), the leukemia cell line K562 and NK-92 cell line were obtained from the American Type Culture Collection (ATCC, Manassas, VA, USA) and cultured in the conditions recommended by ATCC. When the effector (NK-92) and target (breast tissue derived cell lines and K562) cells were cocultured together for assays, the effector complete medium without IL-2 (i.e. Alpha Minimum Essential medium without ribonucleosides and deoxyribonucleosides but with 2 mM L-glutamine and 1.5 g/L sodium bicarbonate supplemented with 12.5% horse serum, 12.5% fetal bovine serum, 0.2 mM inositol, 0.1 mM 2-mercaptoethanol, 0.02 mM folic acid, 100 units/ml penicillin and 100 µg/ml streptomycin) was used. 16–24 hours before effector-target coculture, NK-92 cells were cultured at 500,000 cells/mL in fresh effector complete medium containing 300 U/mL recombinant human IL-2 (PeproTech, Hamburg, Germany). All other media, serum and supplements were from Life Technologies (ThermoFisher Scientific, Waltham, MA, USA), Sigma-Aldrich (St. Louis, MO, USA) or ATCC.

### Transient transfection

Cells were grown to 70–80% confluence and transfected with pCMV3-NCAM1 plasmid (2.5 μg; Cat # HG10673-UT Sinobiological, Wayne, PA, USA) or empty pCMV3 plasmid (2.5 μg; Cat # CV011 Sinobiological) using Lipofectamine 3000 transfection kit (Cat # L3000-015, Invitrogen, ThermoFisher Scientific) according to the manufacturer’s protocol. At 48 hours post-transfection, cells were collected for assessment of transfection efficiency and subsequent analyses.

### Calcein-Acetyoxymethyl (calcein-AM) cytotoxicity assay

Calcein-AM cytotoxicity assay was performed as previously described^37^. Target cells were resuspended in complete medium at a final concentration of 1 × 10^6^ cells/mL and incubated with 15 μM calcein-AM dye (Molecular Probes, Eugene, OR, USA) for 30 min at 37 °C with occasional shaking. After two washes in complete medium, target cells were adjusted to 1 × 10^5^ cells/mL in effector complete medium without IL-2. NK-92 cells were counted in effector complete medium without IL-2 and adjusted to 1 × 10^5^ cells/mL. Then, the effector and target cells were seeded in V-bottom 96-well plate at effector-to-target ratios ranging from 20:1 to 0.62:1, in triplicates. Each well contained from 1 × 10^5^ to 3 × 10^3^ NK-92 in 100uL and 5 × 10^3^ target cells in 50 μl. Six replicate wells for spontaneous (only target cells in effector complete medium without IL-2) and maximum release (only target cells in medium plus 10% Triton X-100) were added. After incubation at 37 °C in 5% CO_2_ for 4 hours, 75 μL of each supernatant was harvested and transferred into new flat-bottom 96-well plate. Samples were measured using FLUOstar Omega microplate reader (excitation filter: 485 ± 9 nm; band-pass filter: 530 ± 9 nm). Data was expressed as arbitrary fluorescent units corresponding to the fluorescent calcein released in the supernatant after lysis of target cells and cytotoxicity percentages were calculated using this formula:

$${Cytotoxicity}\, \% =\frac{{Test}\,{release}-{Spontaneous}\,{release}}{{Maximum}\,{release}-{Spontaneous}\,{release}}\times \,100$$

### Flow cytometry

Cells were washed twice with cold phosphate-buffered saline (PBS) and incubated with PE-conjugated anti-CD56 antibody (1:100 dilution; Cat # 130-090-755 Miltenyi Biotec, Auburn, CA, USA) or APC-conjugated anti-CD56 antibody (1:100 dilution; Cat # 318310 Biolegend, San Diego, CA, USA) or with their respective isotype control antibodies PE-conjugated mouse IgG1 (1:100 dilution; Cat # 130-092-212 Miltenyi Biotec) or APC-conjugated mouse IgG1κ (1:100 dilution; Cat # 400122 Biolegend) for 20 minutes at 4 °C. Then, the cells were washed twice with cold PBS. Data was acquired on BD Accuri™ C6 flow cytometer and analyzed using FlowJo software.

### Degranulation assay

One day before assay, 2.5 × 10^6^ target cells were labeled with 0.5 uM CellTrace CFSE (Carboxyfluorescein succinimidyl ester) (Cat # C34554 ThermoFisher Scientific) and incubated at 37 °C overnight. The next day, CFSE-labeled target cells and NK-92 cells were cocultured at 1:1 effector-to-target ratio at 37 °C for 2 hours and stained with Alexa Fluor® 647-conjugated anti-CD107a antibody (1:100 dilution; Cat # 562622 BD Biosciences) or Alexa Fluor® 647-conjugated mouse IgG1κ isotype control (1:100 dilution; Cat # 557732 BD Biosciences, San Jose, CA, USA). Then, cells were washed twice with cold PBS and analyzed on BD Accuri™ C6 flow cytometer and FlowJo software.

### Active caspase 3 apoptosis assay

One day before assay, 2.5 × 10^6^ target cells were labeled with 0.5 uM CellTrace CFSE and incubated at 37 °C overnight. The next day, CFSE-labeled target cells and NK-92 cells were cocultured at 1:1 effector-to-target ratio at 37 °C for 2 hours. After two washes with cold PBS, cells were fixed using BD Cytofix/Cytoperm solution (Cat # 51-6896KC BD Biosciences). After fixation, cells were washed twice with BD Perm/Wash buffer (Cat # 51-6897KC BD Biosciences) and incubated with PE-conjugated anti-active caspase-3 antibody (Cat # 51-68655X BD Biosciences) for 30 minutes at room temperature. The cells were then washed twice with BD Perm/Wash buffer and analyzed on BD Accuri™ C6 flow cytometer and FlowJo software.

### Immunofluorescence

Cells were grown to 70–80% confluence on cover glass slides (Menzel-Gläser; Braunschweig, Germany), washed twice with PBS and fixed in 4% paraformaldehyde in 0.1 M phosphate buffer (pH 7.4) for 20 minutes at room temperature. The cells were permeabilized for 15 minutes with 0.4% Triton X-100 in PBS (PBST), and blocked with 6% bovine serum albumin (BSA) in PBST for at least 1 hour at room temperature. After removing of the blocking solution, the cells were incubated with mouse anti-human CD56 (NCAM) antibody (1:250 dilution; Cat # 318310 Biolegend) or mouse IgG1κ isotype control (1:250 dilution; Cat # 400122 Biolegend) at 4 °C overnight. The cells were washed three times with tris-buffered saline with 0.3% Tween 20 (TBST) and then incubated with the secondary antibody Alexa Fluor 568-conjugated donkey anti-mouse IgG (1:500 dilution; ThermoFisher Scientific) for 45 minutes at room temperature. The slides were washed three times with TBST, rinsed once with PBS and mounted in Vectashield mounting medium containing 1.5 μg/mL DAPI (Vector Laboratories, Burlingame, CA, USA). The slides were kept protected from light. There were examined and imaged under Zeiss fluorescent microscope and the images were processed using the Adobe Photoshop software.

### Immune synapse formation assay

NK-92 cells were co-cultured with CFSE-labeled target cells in a 96-well plate at 1:1 ratio (150,000 cells/well) for 2 hours at 37 °C. Cell conjugates were then transferred to Poly-L-lysin-coated coverslips placed in 24-well plates. To visualize the interactions between NK-92 and CFSE-labeled target cells, the conjugates were fixed, permeabilized, blocked and stained using the same procedure as in “Immunofluorescence” method above. Alternatively, immune synapse was determined by staining the actin cytoskeleton polymerization at the contacts between NK-92 and target cells (without CFSE labeling) using Alexa Fluor® 488 Phalloidin (Cat # A12379 Molecular Probes, Life technologies). In this alternative method lacking the labeling of target cells, the latter could be distinguished from NK cells based on the size of their nucleus. In fact, in control single cultures (NK alone and target alone) stained with DAPI as in immune synapses involving CFSE-labeled target cells, NK cells were found to have a bigger nucleus than hTERT-HME1 target cells. Therefore, in the cell conjugate forming the immune synapse, the cell that has a bigger nucleus is identified as “NK” and the cell with the smaller nucleus is identified as the target “T”.

The rate of synapse formation was quantified by counting in each representative field or slide the number of NK cells bound to target cells (NK-T) and the number of NK cells unbound to target cells (NK alone), then using the formula:

$${Rate}\,{of}\,{synapse}\,{formation}\,{(}{ \% }{)}=\frac{{number}\,{of}\,{NK}-T\,}{{number}\,{of}\,{NK}-{T}+{number}\,{of}\,{NK}\,{alone}}\times \,{\rm{100}}$$

The localization of CD56 or granzyme B proteins relative to the immune synapse was determined using the primary antibodies mouse anti-CD56 (1:125 dilution, Biolegend) or mouse anti-Granzyme B (1:125 dilution; Cat # MA1-80734 ThermoFisher Scientific), respectively, followed by the secondary antibody Alexa Fluor® 568-conjugated donkey anti-mouse IgG (1:500 dilution; Thermo Fisher Scientific).

### Breast cancer tissue array immunohistochemistry (IHC)

Breast cancer tissues microarray slides were purchased from US Biomax (BC081120c). Each slide contained 110 specimens (100 cases of invasive ductal carcinoma and 10 normal breast tissue). The paraffin-embedded sections (5 μm thickness) were deparaffinized, rehydrated through descending grades of ethanol, and washed with PBS. For antigen retrieval, the slides were incubated in 10 mM Sodium citrate containing 0.05% Tween 20, pH 6.0 for 3 hours at room temperature and rinsed two times with 0.1 M PBS with 0.2% Triton X-100, pH 7.4 (PBST). To block non-specific protein binding, sections were incubated in 6% BSA in PBST overnight at 4 °C, then were incubated for 24 hours at 4 °C with mouse anti-human CD56 (NCAM) antibody (1:250 dilution; Cat # 318310, Biolegend) or with mouse anti-human NKp46 antibody (1:250 dilution; Cat # 331918, Biolegend). The primary antibodies were diluted in 2% BSA in PBST. After rinsing the sections with PBST for three times (15 min each), they were incubated for 2 hours at room temperature with Alexa Fluor 568-conjugated donkey anti-mouse IgG (1:500 dilution; Thermo Fisher Scientific, USA). After three rinses with PBST, Vectashield mounting medium containing 1.5 μg/mL DAPI was added to the slides and then covered with cover slips. The slides were kept away from light and the images were captured using a Zeiss fluorescent microscope.

### Semi-quantitative RT-PCR

Total RNA was extracted from cells using RNeasy Mini Plus Kit (Qiagen, Hilden, Germany) following the manufacturer’s recommendations. The concentration (ng/mL) and the purity of the isolated RNA were measured using NanoDrop 2000 UV-Vis Spectrophotometer (ThermoFisher Scientific). 1 µg RNA from each sample was reversely transcribed using SuperScript™ IV First-Strand Synthesis System (Invitrogen) as per the manufacturer’s instructions. cDNA templates were added to PCR Master Mix (ThermoFisher Scientific) then amplified using specific primers for CD56 (5′-CATCACCTGGAGGACTTCTACC-3′ and 5′-CCAAGGACTCCTGCCCAATG-3′) or GAPDH housekeeping gene (5′-ACGACCACTTTGTCAAGCTCATTTC-3′ and 5′-GCAGTGAGGGTCTCTCTCTTCCTCT-3′). The PCR reactions were carried in a thermal cycler with the following protocol: an initial denaturation step for 3 minutes at 95 °C, followed by 35 cycles of amplification (95 °C for 30 seconds, 59 °C for 30 seconds, and 72 °C for 45 seconds), and a final extension step for 10 minutes at 72 °C. PCR products were separated on 2% agarose gel supplemented with ethidium bromide. Images were visualized with BioRad Chemidoc XRS system and analyzed by ImageJ software.

### RNA-seq sample preparation, library construction and sequencing

Total RNAs extracted from cells using RNeasy Mini Plus Kit were checked on Agilent Bioanalyzer 2100 system to measure the integrity of the samples then on Thermo QuBit 2.0 Flourimeter to measure their concentration. All samples that have good quality were taken forward to prepare the libraries for the sequencing. Libraries were prepared using TruSeq mRNA Stranded Library Prep Kit (Illumina Inc) by following the manufacturer’s protocol. Briefly, mRNAs were captured by Oligo dT selection and fragmented. Fragments were then subjected to end filling, A-tailing before ligating them with Illumina adaptor. Ligated fragments underwent 12 rounds of PCR cycles to amplify the fragments in order to have enough sequencing libraries. The quality control analyses of sequencing libraries were performed again by QuBit for quantification and on Agilent technologies 2100 Bioanalyzer chip to measure the quality and to measure the library size. The quality control passed libraries were sequenced on an Illumina HiSeq 4000 instrument by Illumina Sequencing-By-Synthesis (SBS) method. 2.5 nM were loaded for each library and 12 samples were loaded on each lane of the HiSeq 4000 flow cell. Paired end data were generated for 300 cycles and each sample having a minimum of 25 million cluster reads.

### Bioinformatic analyses

Quality control (QC) for the raw reads was performed with the FastQC version 0.11.5 as per the FastQC-project parameter recommendations (http://www.bioinformatics.babraham.ac.uk/projects/fastqc/). Besides, RNA-Seq analysis workflow was designed and conducted through the CLC Biomedical Genomics Workbench v5.0.1 (QIAGEN Aarhus, Denmark). The first step incorporated a QC enhancement which included length (less than 60 bp) and quality trimming of the short sequence reads. Then, the paired-end short fragments reads were assembled, mapped and annotated against the Homo Sapiens RefSeq GRCh37 genome and transcripts build. Next, the gene expression profile was estimated for each gene using the total count where the reported level is the number of reads that map to the exons of that gene. For the hierarchical clustering of the samples’ expression patterns similarity over their genes, the Create Heat Map tool was used. Candidate genes are normalized by applying the trimmed mean of M-values normalization method (TMM). Then, the clustering algorithm was defined by the Euclidean distance measurement and the complete linkage.

### Statistical analysis

Statistical significance of differences between experimental groups was performed by Student’s t-test and relationships between CD56 expression and clinical parameters were identified by chi-square (χ^2^) test, using the GraphPad Prism 7 software (San Diego, CA, USA). Differences were considered significant at confidence levels greater than 95% (P < 0.05).

## Supplementary information

Supplementary information

## Data Availability

RNA sequencing datasets generated during the current study are available in the Zenodo repository (10.5281/zenodo.2784989, 10.5281/zenodo.2813842 and 10.5281/zenodo.2918674). The other datasets are available in the supplementary information of this article or from the corresponding author on reasonable request.
