# Supplementary material for: CD56 expression in breast cancer induces sensitivity to natural killer-mediated cytotoxicity by enhancing the formation of cytotoxic immunological synapse
Source: Sci Rep. 2019 Jun 19;9:8756. doi: 10.1038/s41598-019-45377-8 (PMC6584531; doi:10.1038/s41598-019-45377-8)

Supplementary information

Manuscript title: CD56 expression in breast cancer induces sensitivity to natural killer-mediated cytotoxicity by enhancing the formation of cytotoxic immunological synapse

Authors: Ghina Taouk, Ola Hussein, Moussa Zekak, Ali Abouelghar, Yasser Al-Sarraj, Essam M. Abdelalim and Manale Karam

Descriptive captions for supplementary materials

Supplementary Figure 1: Potential expression of CD56 adhesion molecule by normal, precancerous and cancerous cells of mammary origin**.** (A) Analysis of NK-92 degranulation following coculture with breast cancer target cells, at NK-92:target ratio of 1:1, by flow cytometry using CD107a and CD56 co-staining. Results show that the phenotypic marker of NK cells (CD56) cannot be used to discriminate between NK-92 (CD56-positive) and breast cancer cells (expected to be CD56-negative) as NK-92-senstive hTERT-HME1 and BT549 seem to express this adhesion molecule (> 95% of the coculture cells were CD56-positive). However, NK-92-resistant BT20 and T47D are found to be CD56-negative. (B) Differential CD56 (i.e. NCAM1) gene expression analysis between the different NK-92-sensitive and resistant breast precancerous and cancerous cell lines by RNA sequencing. The heatmap shows differential expression of NCAM1 and ESR1 genes between the indicated cells lines. Deep red indicates higher expression and deep blue indicates lower expression. ESR1 gene encoding the estrogen receptor α (ERα) was used as control and found to be specifically expressed in both replicates of the ERα-positive breast cancer cell lines (MCF-7, T47D, HCC1500 and BT474). Results show high expression of CD56 in the NK-92 sensitive cell lines (hTERT-HME1 and BT549) and the PMEC cell line but very low (HCC1954) and no expression (MCF-7, BT474, BT20, MDA-MB-231, SKBR3, T47D, HCC2500 and K562) in the NK-92-resistant breast cancer cell lines.

Supplementary Figure 2: Analysis of CD56 expression in breast cancer tissues by immunohistochemistry. Breast carcinoma microarray (BC081120c - US Biomax) containing 110 specimens (100 cases of invasive ductal carcinoma [samples A1-10 to J1-10] and 10 normal breast tissue [samples K1-10]) was prepared and stained for CD56 and DAPI by

immunohistochemistry, as indicated in the "material and methods" section. (A) Images for each sample. (B) Graph depicting the distribution of high (70-100% of CD56-positive cells per tissue), moderate (30-69% of CD56-positive cells per tissue) and low (0-29% of CD56-positive cells per tissue) CD56 expression levels for malignant versus normal breast tissues. Thus, CD56 can be expressed in normal and cancer tissues of mammary origin.

Supplementary Figure 3: Prognostic value of CD56 and NKp46 mRNA expression in breast cancer patients. The correlation between CD56 mRNA expression (probe ID: 227394_at) (A) or NKp46 mRNA expression (probe ID: 207860_at) (B) and relapse-free survival (RFS) and overall survival (OS) in breast cancer patients was analyzed by using the KM plotter database (http://kmplot.com/analysis/). Breast cancer samples were split into high and low expression groups according to the lower quartile value and the two patient cohorts were compared by Kaplan-Meier survival plots. The hazard ratio (HR) with 95% confidence intervals and log rank P values were calculated. High mRNA expression of CD56 significantly associates with better RFS but not OS. High mRNA expression of NKp46 significantly associates with better RFS and OS. (C) Differential NKp46 (i.e. NCR1) gene expression analysis between the NK-92 cells and different NK-92-sensitive and resistant breast precancerous and cancerous cell lines by RNA sequencing. The heatmap shows differential expression of NCR1 and ESR1 genes between the indicated cells lines. Deep red indicates higher expression and deep blue indicates lower expression. ESR1 gene was used as control and found to be specifically expressed in both replicates of the ERα-positive breast cancer cell lines (MCF-7, T47D, HCC1500 and BT474). Results show a specific high expression of NKp46/NCR1 in the NK-92 cells but not in breast precancerous and cancerous cell lines.

Supplementary Figure 4: Analysis of NKp46 expression in breast cancer tissues by immunohistochemistry. Breast carcinoma microarray (BC081120c - US Biomax) containing 110 specimens (100 cases of invasive ductal carcinoma [samples A1-10 to J1-10] and 10 normal breast tissue [samples K1-10]) was prepared and stained for NKp46 and DAPI by immunohistochemistry, as indicated in the “material and methods” section. The results show that NKp46 is not expressed in normal and cancer tissues of mammary origin.

Supplementary Figure 5: Differential expression analysis of NK regulating genes between the different NK-92-sensitive and resistant breast precancerous and cancerous cell lines by RNA sequencing. (A) The heatmap shows differential expression of all genes encoding ligands for NK regulatory receptors (activating and inhibitory) that are known to date (Abouelghar et al 2018 Oncotarget), between the indicated cells lines. Deep red indicates higher expression and deep blue indicates lower expression. Among these ligands, only CD70, CD72 and COL3A1 were found to have an expression profile comparable to that of CD56 in breast-derived cell lines. CD70 and CD72 are ligands for the NK-activating receptors CD27 and SEMA4D, respectively. COL3A1 is a NK-inhibitory ligand. (B) The heatmap shows differential expression of CD27 and SEMA4D NK-activating receptors between the different cell lines. SEMA4D but not CD27 seems to be expressed in NK-92 cell line.

Supplementary Figure 1

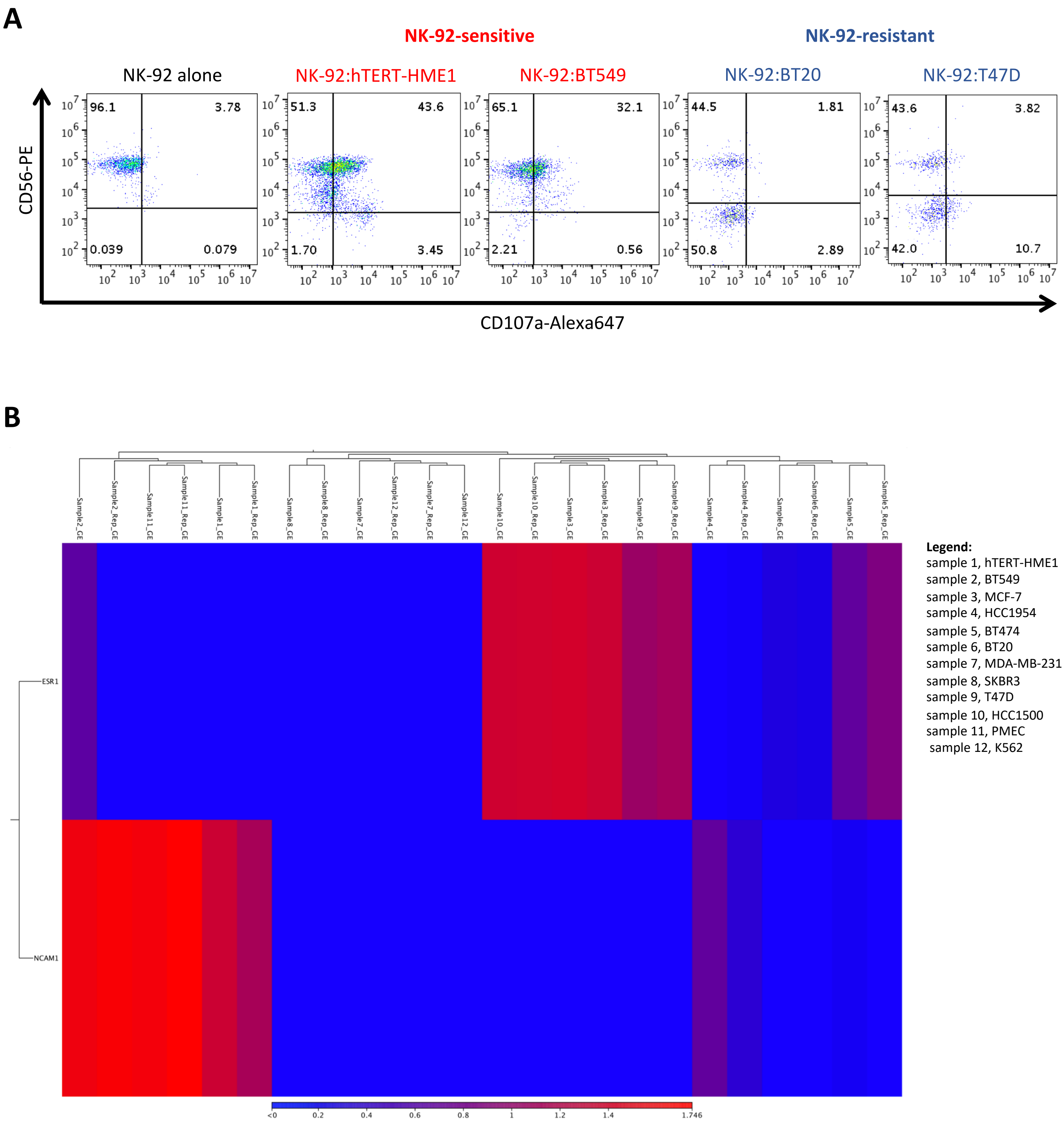

Supplementary Figure 2

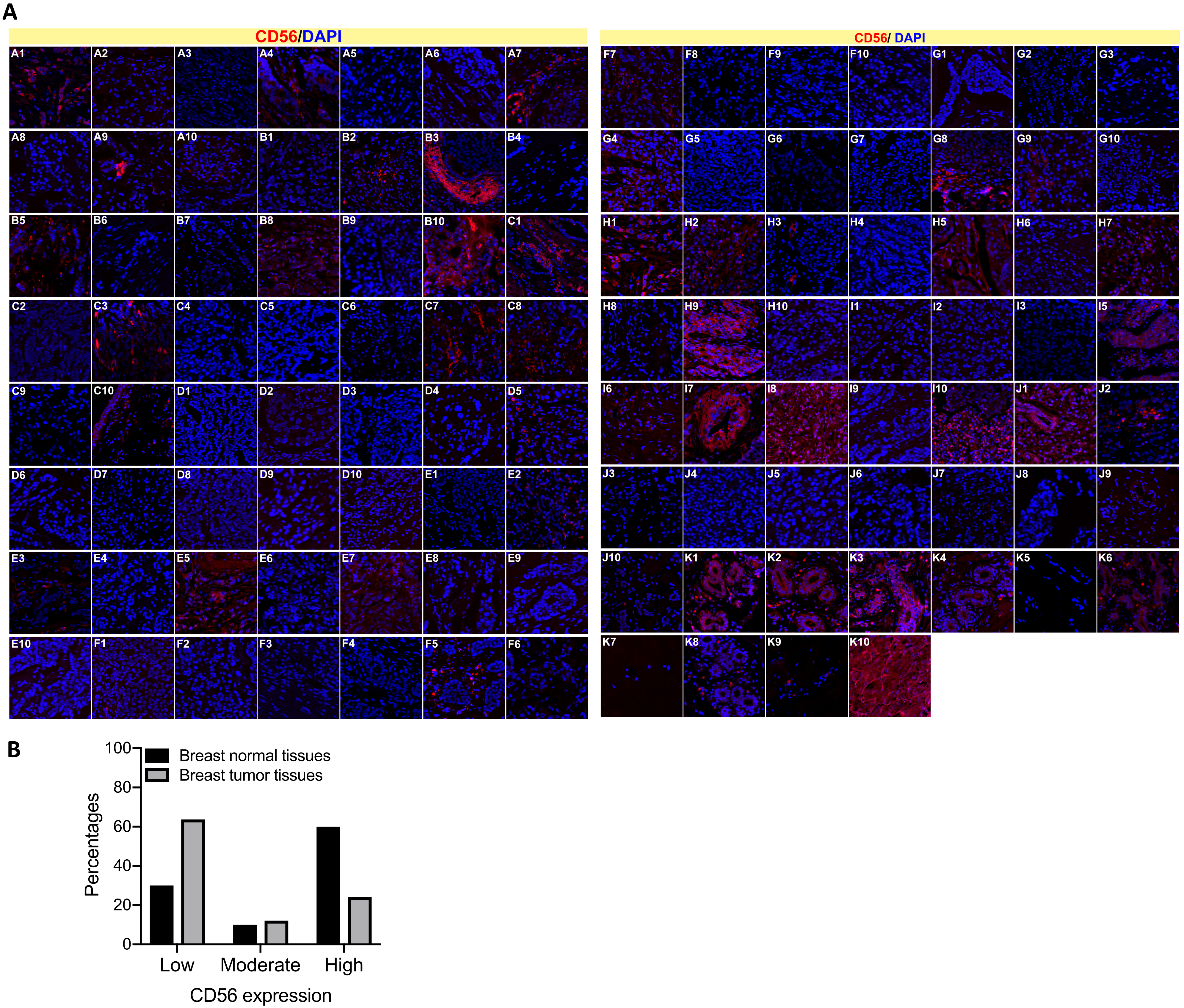

Supplementary Figure 3

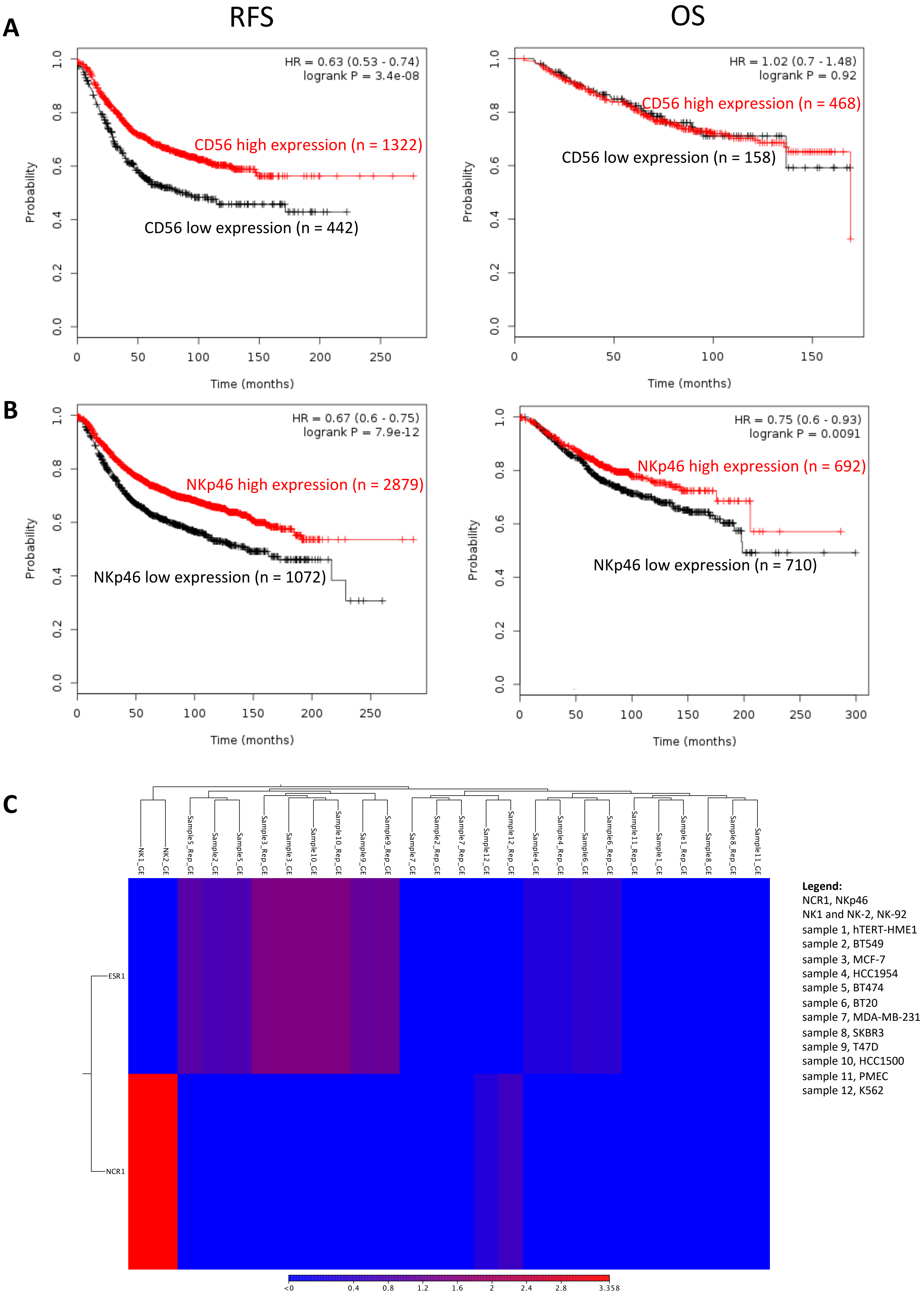

Supplementary Figure 4

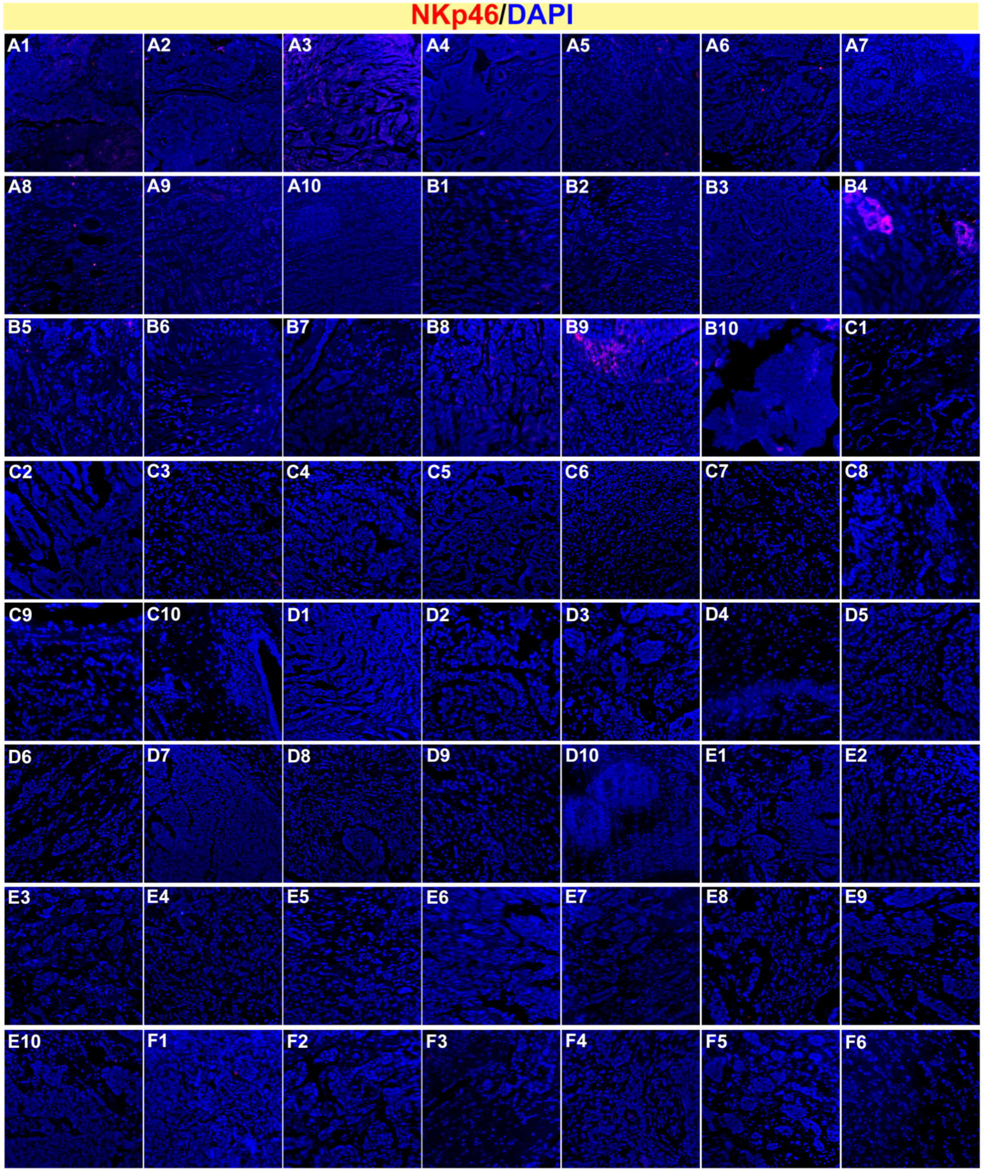

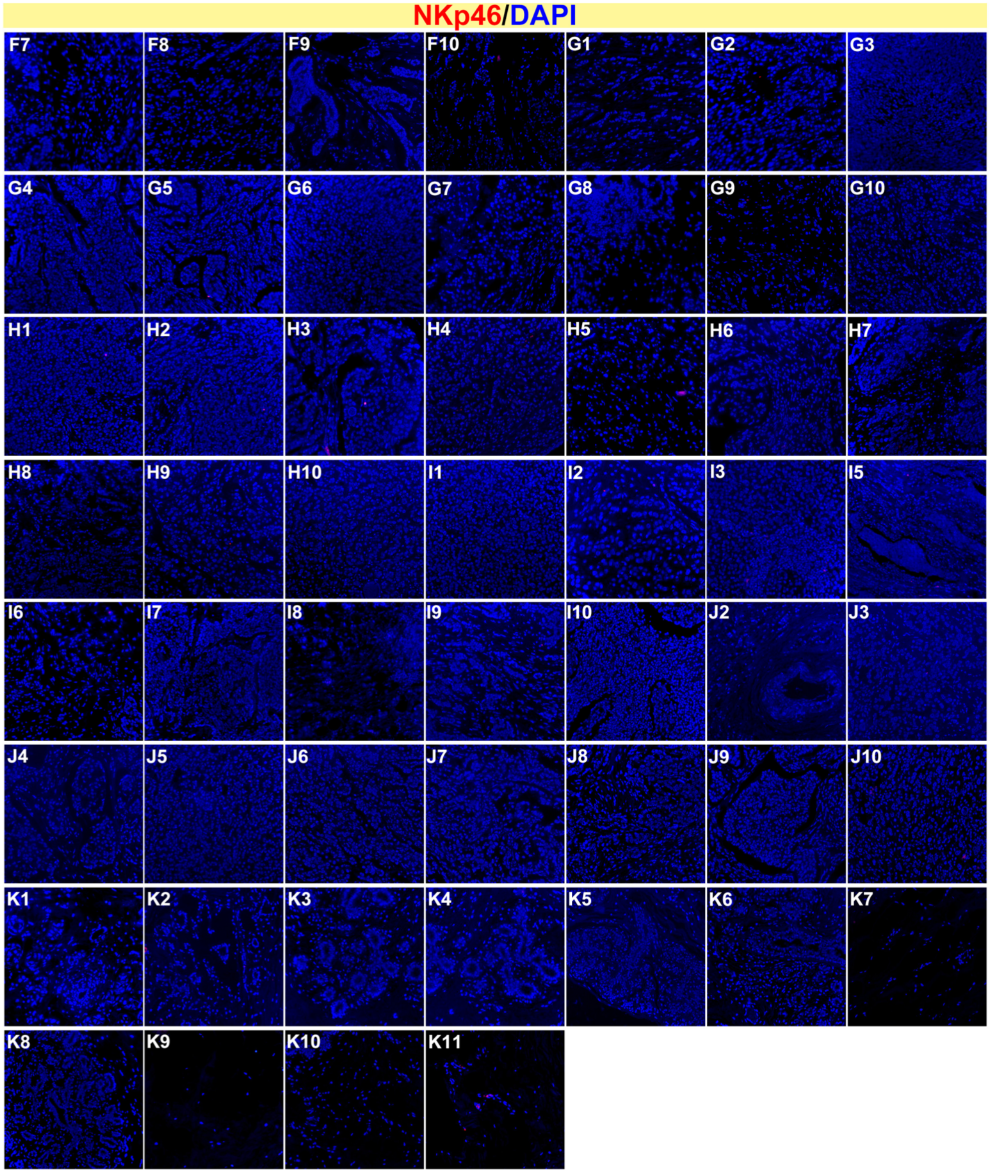

Supplementary Figure 5

A

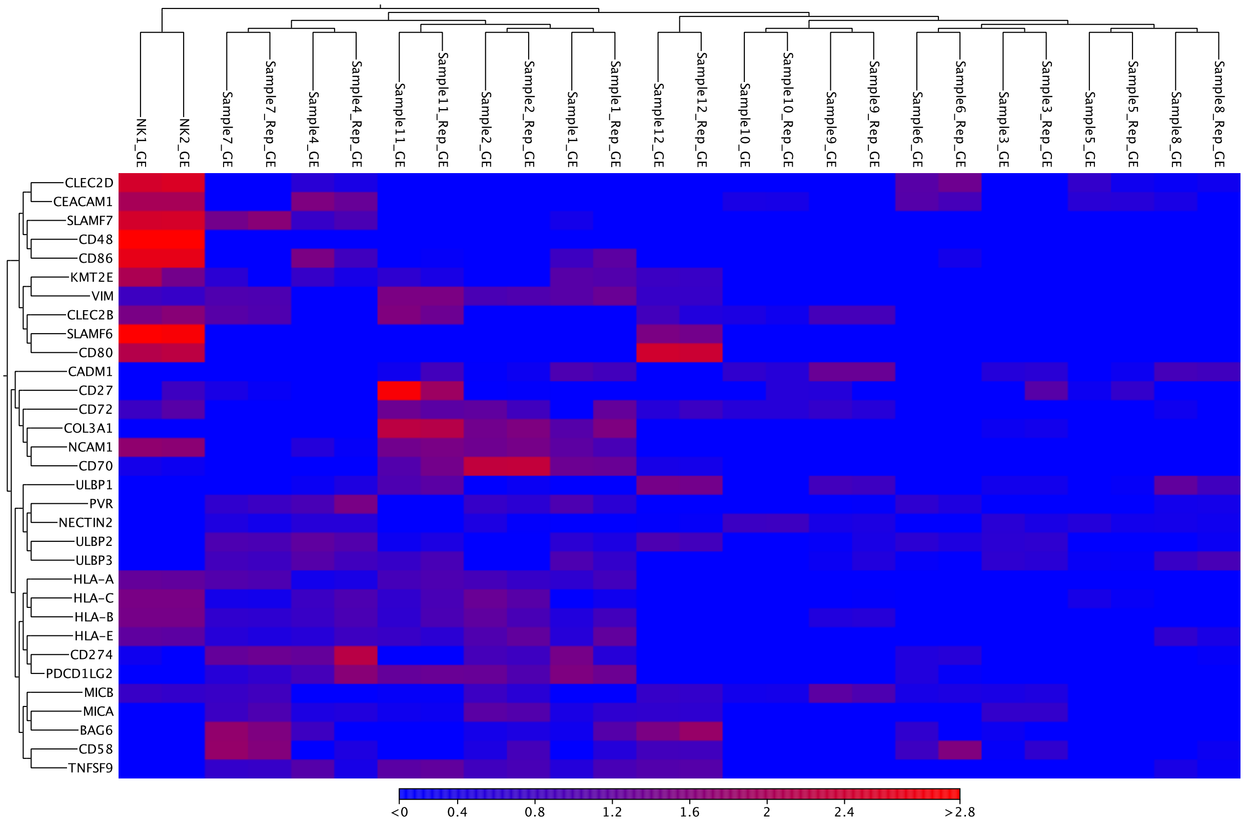

Legend:
NK1 and NK2, NK-92
sample 1, hTERT-HME1
sample 2, BT549
sample 3, MCF-7
sample 4, HCC1954
sample 5, BT474
sample 6, BT20
sample 7, MDA-MB-231
sample 8, SKBR3
sample 9, T47D
sample 10, HCC1500
sample 11, PMEC
sample 12, K562

B

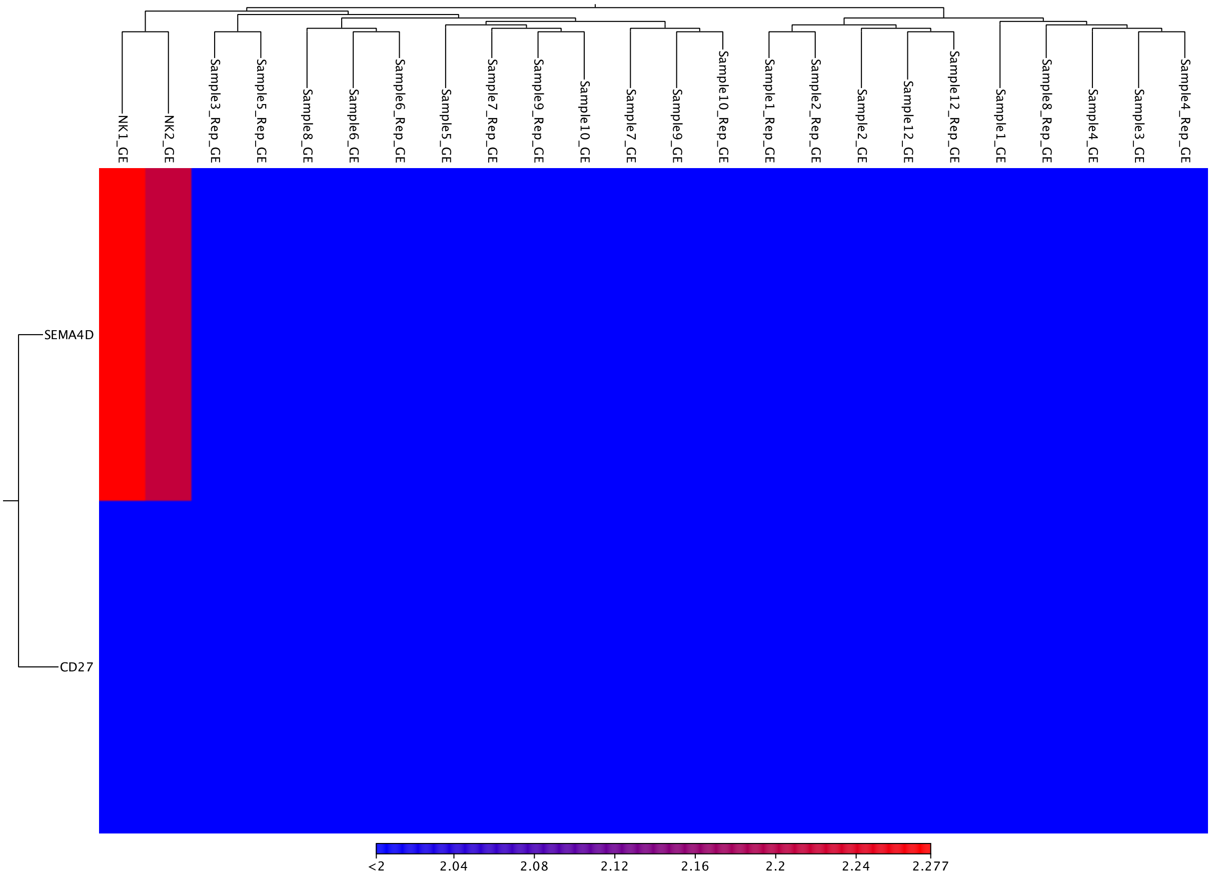

Full-length gels – Fig 3A

Fig 3A - upper gel CD56

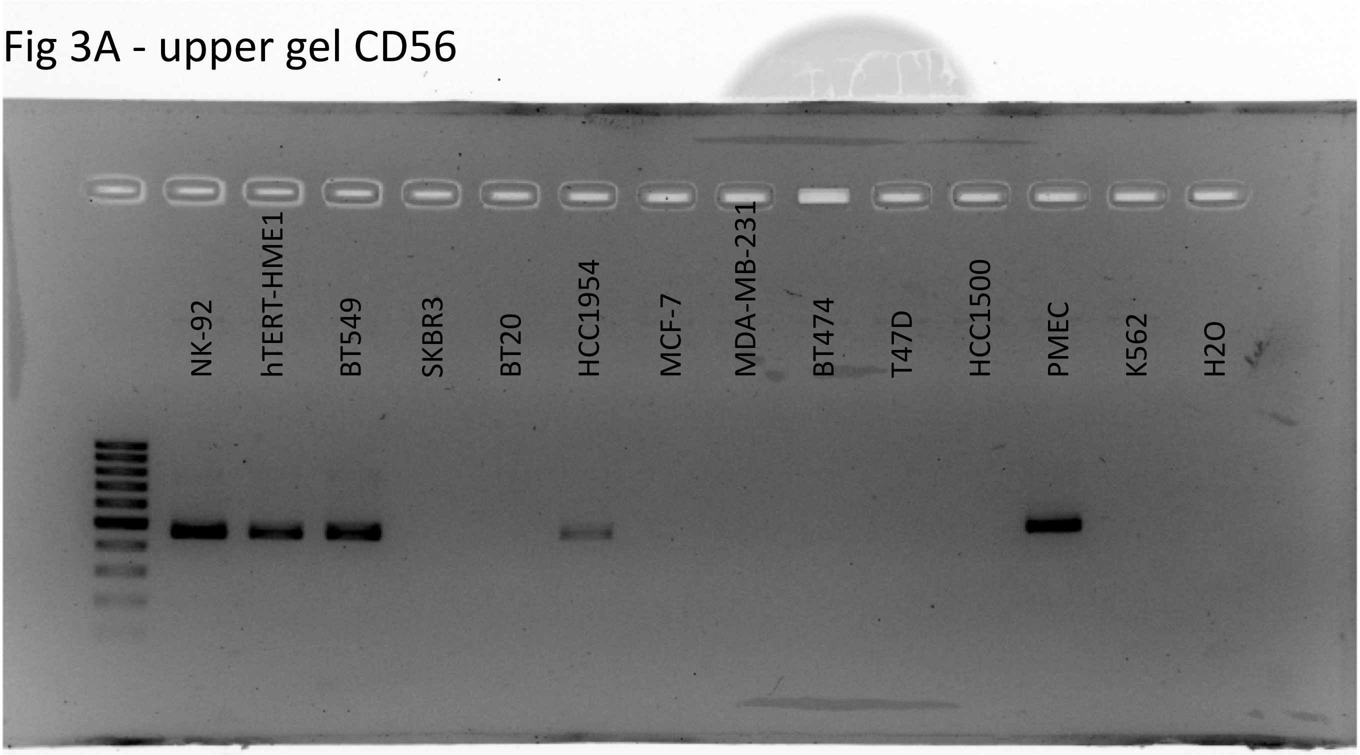

Fig 3A - lower gel GAPDH

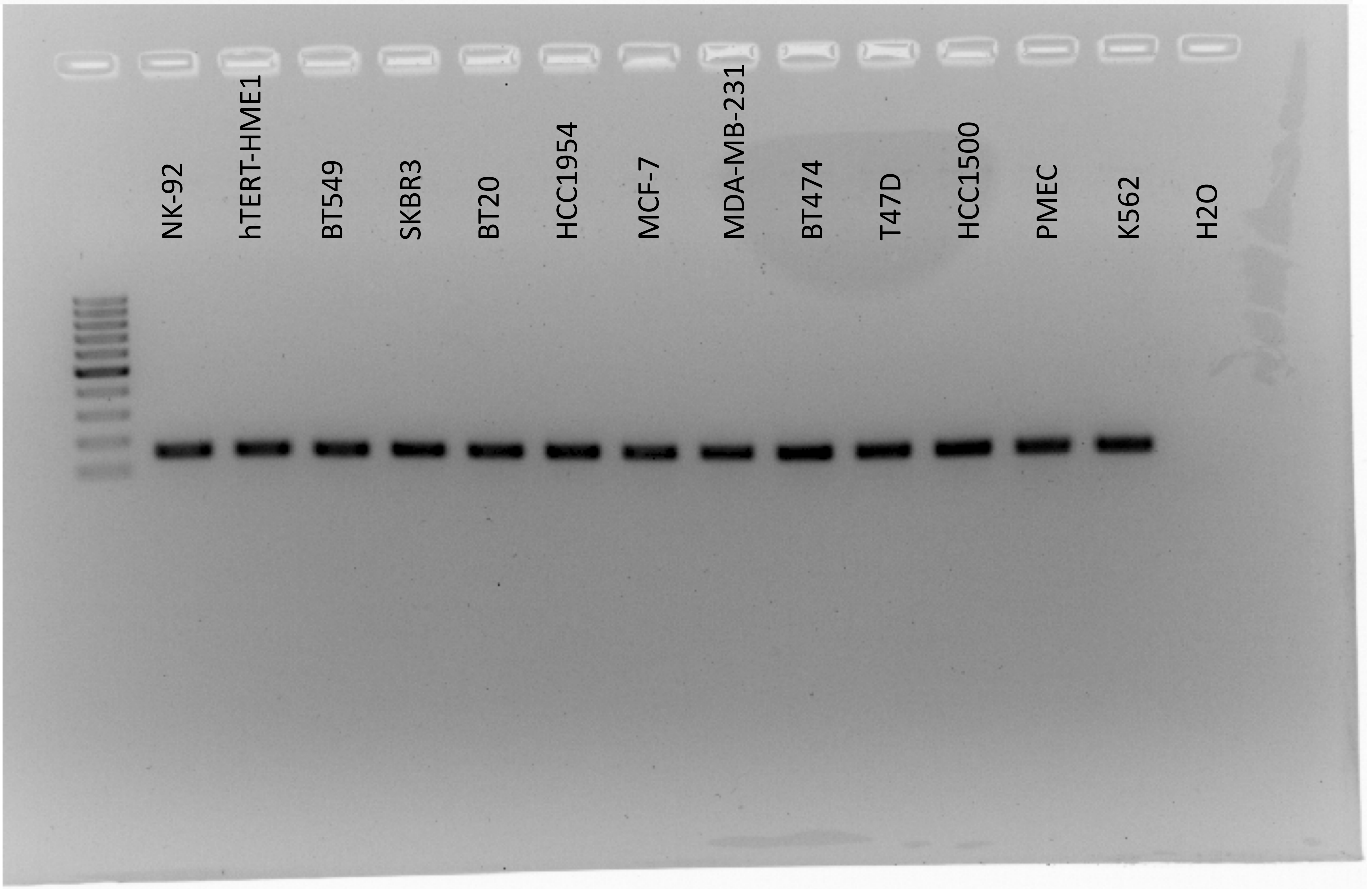

Supplement: Supplementary file 1 — Supplementary information [file 41598_2019_45377_MOESM1_ESM.pdf]
